# Functional and Technical Aspects of Self-management mHealth Apps: Systematic App Search and Literature Review

**DOI:** 10.2196/29767

**Published:** 2022-05-25

**Authors:** Lyan Alwakeel, Kevin Lano

**Affiliations:** 1 Department of Informatics, King’s College London London United Kingdom; 2 College of Computers & Information Technology, University of Tabuk Tabuk Saudi Arabia

**Keywords:** mHealth, mobile health apps, mobile apps, apps, systematic literature review, SLR, apps, Mobile App Rating Scale, MARS, smartphone, iOS, Android, mobile phone

## Abstract

**Background:**

Although the past decade has witnessed the development of many self-management mobile health (mHealth) apps that enable users to monitor their health and activities independently, there is a general lack of empirical evidence on the functional and technical aspects of self-management mHealth apps from a software engineering perspective.

**Objective:**

This study aims to systematically identify the characteristics and challenges of self-management mHealth apps, focusing on functionalities, design, development, and evaluation methods, as well as to specify the differences and similarities between published research papers and commercial and open-source apps.

**Methods:**

This research was divided into 3 main phases to achieve the expected goal. The first phase involved reviewing peer-reviewed academic research papers from 7 digital libraries, and the second phase involved reviewing and evaluating apps available on Android and iOS app stores using the Mobile Application Rating Scale. Finally, the third phase involved analyzing and evaluating open-source apps from GitHub.

**Results:**

In total, 52 research papers, 42 app store apps, and 24 open-source apps were analyzed, synthesized, and reported. We found that the development of self-management mHealth apps requires significant time, effort, and cost because of their complexity and specific requirements, such as the use of machine learning algorithms, external services, and built-in technologies. In general, self-management mHealth apps are similar in their focus, user interface components, navigation and structure, services and technologies, authentication features, and architecture and patterns. However, they differ in terms of the use of machine learning, processing techniques, key functionalities, inference of machine learning knowledge, logging mechanisms, evaluation techniques, and challenges.

**Conclusions:**

Self-management mHealth apps may offer an essential means of managing users’ health, expecting to assist users in continuously monitoring their health and encourage them to adopt healthy habits. However, developing an efficient and intelligent self-management mHealth app with the ability to reduce resource consumption and processing time, as well as increase performance, is still under research and development. In addition, there is a need to find an automated process for evaluating and selecting suitable machine learning algorithms for the self-management of mHealth apps. We believe that these issues can be avoided or significantly reduced by using a model-driven engineering approach with a decision support system to accelerate and ameliorate the development process and quality of self-management mHealth apps.

## Introduction

Self-management mobile health (mHealth) apps use mobile devices for health services and offer a sustainable means of enhancing self–health care management to achieve wellness goals, such as health monitoring, disease detection, behavior change, and emotion management, enabling individuals to independently manage their lives and activities and make appropriate decisions. They comprise different categories, ranging from simple apps for water intake tracking to complex apps that can adapt to individuals’ lives based on their activities. The power of self-management mHealth apps has increased with the use of built-in mobile technologies (eg, cameras, GPS, and accelerometers) and machine learning (ML) algorithms to create intelligent mobile apps. Such apps are characterized by personalized services and recommendations and by the automatic logging and recognition of individuals’ behaviors and activities.

Although mobile apps are extensively used for self–health care management, the ongoing development of mobile device technologies and programming languages has increased the need for mobile app solutions to keep pace with development practices. Developing high-quality intelligent self-management mHealth apps requires substantial knowledge of mobile programming languages, app architectures, design patterns, and latest technologies. Considerable time is required from researchers and developers to learn and master such knowledge because of the different characteristics, requirements, and components of each mobile app. Furthermore, many challenges and issues may arise during app development. Therefore, we conducted a comprehensive systematic literature review (SLR) and evaluation of existing self-management mHealth apps that focus on self–health care management, based on a formal protocol, to analyze their characteristics and current challenges, including infrastructure, functionalities, user interface (UI) components, screen navigation, services and technologies, security and authentication, use of architectures and patterns, evaluation, and issues to provide a guide for self-management mHealth app infrastructure to facilitate further development.

Although several SLRs on mHealth apps have been previously conducted, our SLR is distinguished by providing engineering perspectives on 3 different sources: research papers, app stores, and GitHub repositories. The SLR involved the analysis and synthesis of empirical evidence by software engineering researchers to help researchers and developers in three main aspects: (1) to identify the characteristics and challenges of existing self-management mHealth apps, (2) to understand the differences and similarities of existing self-management mHealth apps and find the gap between research papers and commercial and open-source apps, and (3) to suggest future research directions based on gaps identified in the domain. The main contributions of this study are as follows:

- The definition of an SLR protocol following the SLR guidelines by Kitchenham and Charters [1], which is based on a wide range of literature and the selection of 52 research papers, 42 app store apps, and 24 open-source apps as primary studies
- Extraction, analysis, synthesis, and reporting of empirical evidence from the selected primary studies
- Provision of guidance for researchers and developers to deeply understand the characteristics and challenges of self-management mHealth apps
- Suggestions of solutions to overcome the limitations of existing self-management mHealth apps

The principal aim of this SLR was to obtain a detailed view of existing self-management mHealth apps used for self–health care management. A specific objective was to better characterize the functional and technical aspects of these apps.

## Methods

### Study Design

**Figure 1:**
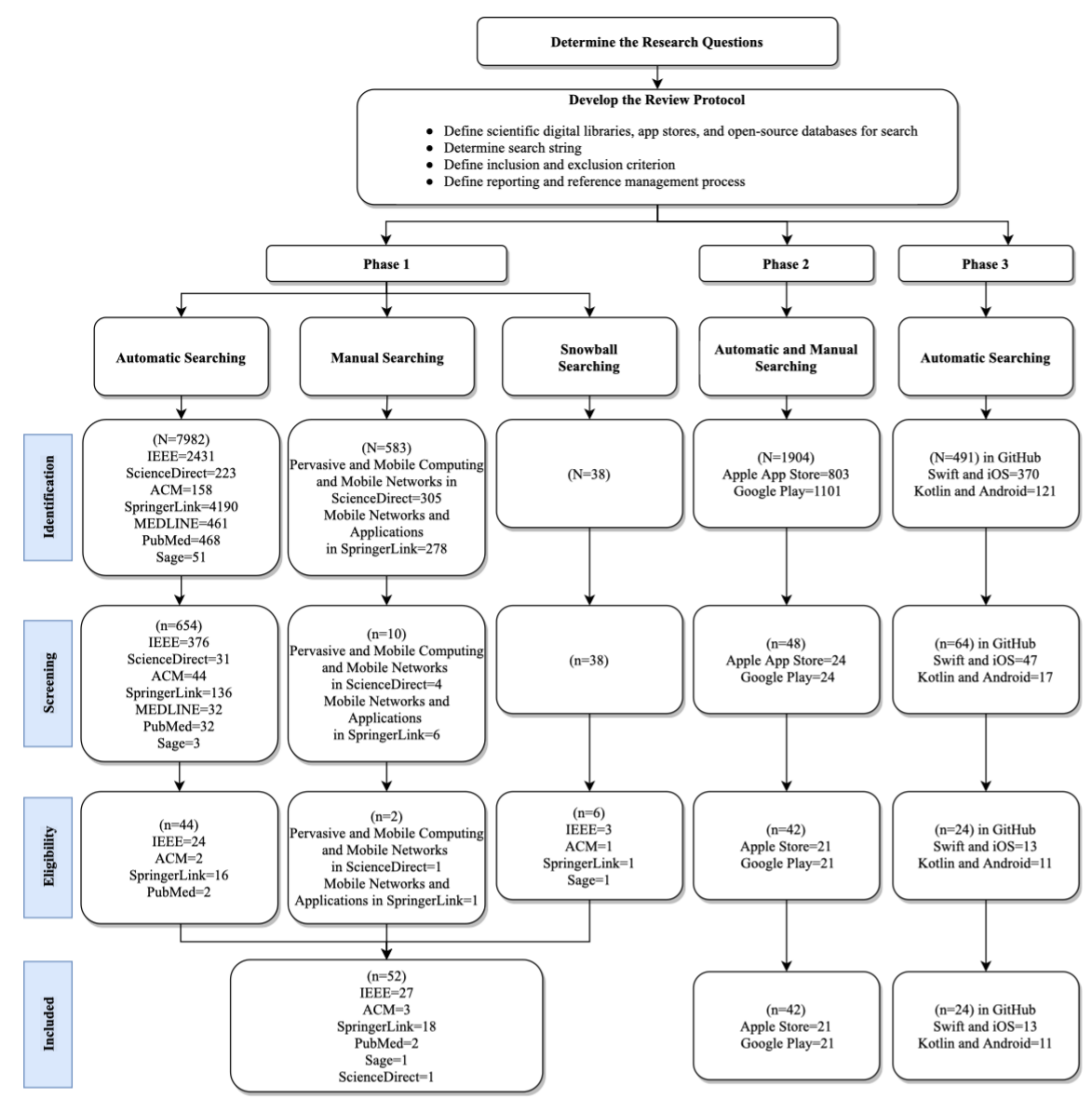
Process diagram for this systematic literature review. mHealth: mobile health.

This review presents the main characteristics and challenges of the self-management of mHealth apps. We targeted apps that use mobile devices for the self–health care management of general users. The study process diagram is shown in Figure 1 where it is divided into 3 main phases for analyzing apps that exist in the knowledge base:

1. Phase 1: a comprehensive review of existing research papers on self-management mHealth apps in digital libraries
2. Phase 2: an exploration of self-management mHealth apps available through Britain’s Apple App Store and Android Google Play, as well as an evaluation of the selected apps using the Mobile App Rating Scale (MARS) [2]
3. Phase 3: analysis of open-source apps available on GitHub based on specific criteria and an automatic tool (ie, SonarCloud; SonarSource SA) [3]

### Review Methodology

This section presents related literature in the knowledge base, which forms the foundation of our SLR. The SLR follows the Kitchenham and Charters [1] guidelines, which divide the review of each phase into 3 main stages of the review: planning, conducting, and reporting. All the stages were prepared by the first author and revised by the second author. The following subsections outline the steps that were followed.

### Research Questions

#### Overview

To investigate and deduce empirical evidence of existing self-management mHealth apps, we determined 2 key research questions (RQs):

- RQ1: What are the main characteristics of current self-management mHealth apps?
- RQ2: What are the challenges and issues faced by current self-management mHealth apps?

#### Detailed Explanation of the RQs

##### The Characteristics of Self-management mHealth Apps

We determined some characteristics of self-management mHealth apps that we were interested in monitoring because of their importance in the development of mobile apps. We collected these characteristics based on their availability during each phase (Textbox 1).

**Characteristics of self-management mobile health apps.**

**Crucial functionalities**

- Each app comprises several components that define its functionality. We have summarized the main functionalities of the reviewed apps.

**User interface components**

- We determined the user interface components used by the users for interaction.

**Navigation and structure**

- Here, we explored the apps’ organization and methods of navigating the app screens.

**Services and technologies**

- We determined the remote and local services that are external to the assigned app but handled by it, such as machine learning algorithms; built-in technologies; and access to other apps, frameworks, and libraries.

**Security features**

- We were interested in defining the security aspects and authentication mechanisms used in the self-management of mobile health (mHealth) apps.

**Architectures and patterns**

- There are different architectures and patterns for building apps, such as client-server, model-view-controller, and view-interactor-presenter-entity-router. In this study, we determined the most commonly used architectures and patterns. Furthermore, we specified the architecture for implementing machine learning, including web-based inference, offline inference, or both.

**Logging mechanisms**

- We determined the apps’ method for logging data, either manually or automatically.

**Development approach**

- We explored the main development approaches which developers use to construct apps.

**Operating system and programming language**

- We identified the operating system and programming languages used in the reviewed apps.

**Evaluation**

- Here, we were concerned with the techniques for evaluating self-management mHealth apps used by researchers in phase 1. Furthermore, we evaluated the selected apps using Mobile App Rating Scale [2] and SonarCloud (SonarSource SA) tools to assess their quality in phases 2 and 3. We used the Mobile App Rating Scale in phase 2, which is a reliable tool developed by an expert panel for evaluating the quality of mHealth apps and comprises an initial section for gathering general and technical information about the assigned app and 5 specific sections: engagement, functionality, aesthetics, information quality, and subjective quality. Each section has a group of items that can be scored from 1 (inadequate) to 5 (excellent). These scores are used to calculate the mean score for each section. Finally, the average values of the mean of the first 4 sections (ie, engagement, functionality, aesthetics, and information quality) are calculated to obtain the final measurements of app quality. All apps were evaluated and compared to find the differences between platform versions. In phase 3, we used a web-based service (SonarCloud) for static code analysis, as well as manual exploration to identify app characteristics.

##### The Challenges and Issues of mHealth Apps

We identified the limitations and challenges faced by researchers and developers when developing self-management mHealth apps. In phase 1, we summarize the researcher’s challenges and issues. In phases 2 and 3, we identified potential issues that can affect the quality of apps using MARS and SonarCloud, such as design issues, bugs, code smell, and duplication.

### Search Strategy

#### Overview

A search strategy was followed to explore the literature that could help answer the RQs. It comprises 3 main stages: defining search strings, selecting data sources, and searching the data sources. As previously mentioned, the review is divided into 3 independent phases, each of which has separate data sources and search strings. In the first phase, we followed the quasi–gold standard [4] approach, including manual and automatic searches, as well as snowballing. The second phase included manual and automatic searches, whereas the third phase was limited to automatic searches.

#### Defining Search Strings

The search string of the first phase was defined by combining synonymous terms using *OR* and *AND*. On the basis of our RQs, 5 search strings were identified, as listed in Textbox 2.

In the second phase, an extensive search was performed in Britain’s Apple Store and Google Play from August 16 to August 21, 2020. Although the search was limited to Britain’s app stores because of the requirement to specify the user’s location, most of the selected apps were available in other stores. We started the automatic search by applying the following search string: *mHealth*, *Healthcare*, and *Health*. However, many unrelated apps were identified. Consequently, we changed the search strategy to a manual exploration of the *Top Free App* under the *Health and Fitness* category. These apps are free to download, although many require a monthly payment or upgrade payment to access all features.

In the third phase, we used the search strings *mHealth*, *Healthcare*, and *Health* in GitHub from October 5 to October 9, 2020, to identify open-source mHealth apps.

**Search string used for digital libraries.**

**Search strings**

1. “mHealth” AND (“app” OR “application”).
2. “mobile” AND “health” AND (“app” OR “application”).
3. “Personal” AND “Mobile” AND “healthcare” AND (“app” OR “application”).
4. “Self-management” AND “healthcare” AND (“app” OR “application”).
5. “Smartphone” AND “health” AND (“app” OR “application”).

#### Data Sources

To find studies related to phase 1, we followed an automatic search using the following digital libraries: IEEE Xplore, ScienceDirect, ACM Digital library, SpringerLink, MEDLINE, PubMed, and Sage. To complement the automatic search, a manual search was conducted on relevant journals, including Pervasive and Mobile Computing and Mobile Networks and Mobile Networks and Applications. To find as many studies as possible, we used the snowballing strategy to gather additional studies from the reviewed studies. We used Google Scholar to search for further studies identified from snowballing. In phase 2, we used an automatic and manual search within the following official digital British app stores: Apple iPhone (App Store) and Android (Google Play). In the third phase, we applied an automatic search on GitHub to download open-source apps.

#### Search Process in Data Sources

To identify all related studies, search strings were applied to the selected digital libraries. Initially, 7982 results were retrieved within the chosen search string as follows: 2431 (30.46%) papers from IEEE, 223 (2.79%) papers from ScienceDirect, 158 (1.98%) papers from ACM, 4190 (52.49%) papers from SpringerLink, 461 (5.78%) papers from MEDLINE, 468 (5.86%) papers from PubMed, and 51 (0.64%) papers from Sage. The results were filtered based on the title and abstract, and duplicated and unrelated papers were removed. Then, of the 7982 papers, 654 (8.19%) were downloaded for examination. According to the inclusion and exclusion criteria, of the 654 studies, 44 (6.7%) studies were included from the automatic search. Furthermore, we found 2 studies from the manual search and 6 studies from snowballing. Thus, we collected 52 studies from automatic and manual searches, as well as snowballing. Manual search studies were from ScienceDirect and SpringerLink. For the 6 studies from snowballing, 3 (50%) papers were from IEEE, and 1 (17%) paper each was from SAGE, ACM, and SpringerLink.

The systematic search process was applied to identify mobile apps for the general population in phase 2. Initially, 1904 apps were retrieved with the chosen search strategy: 803 (42.17%) apps from Apple’s App Store and 1101 (57.83%) apps from Android’s Google Play. The results were filtered based on the inclusion and exclusion criteria and availability in both app stores. After removing duplicate apps, we downloaded and explored 100% (48/48) of apps on each platform, iOS and Android. A total of 48 apps were reviewed for further refinement after applying the inclusion and exclusion criteria. Then, 6% (3/48) of apps from each store were removed as they were not used for self–health care management. Therefore, of the 42 apps, the final number of apps included on each platform was 21 (50%).

In phase 3, the systematic search process was applied to GitHub to identify self-management mHealth apps for the general population using the Swift and Kotlin programming languages. Initially, 491 apps were retrieved with the chosen search strategy as follows: 370 (75.4%) apps with Swift programming language and 121 (24.6%) apps with Kotlin language. After removing duplicates and unrelated titles, of the 491 apps, we obtained 64 (13%). These apps were downloaded and analyzed based on the inclusion and exclusion criteria. After applying the inclusion and exclusion criteria, of the 64 apps, we obtained 24 (38%). These apps were analyzed using the SonarCloud tool, including 13 (54%) iOS and 11 (46%) Android apps.

#### Inclusion and Exclusion Criteria

Textbox 3 presents the inclusion and exclusion criteria that were applied to the downloaded papers that resulted from the manual, automatic, and snowballing search of digital libraries, as well as apps that resulted from digital app stores and GitHub.

**The inclusion and exclusion criteria of self-management mobile health apps.**

**Phase 1**

- Inclusion criteria
- Papers presenting the design and implementation of either or both Android and iOS self–health care management apps
- English peer-reviewed papers published from 2008, the year the App Store was announced [5], to 2020
- The most recent and complete version of a study if it had multiple versions
- Exclusion criteria
- Papers presenting theoretical research without implementation
- Papers describing apps for wearable devices
- Papers targeting children or people with special needs
- Short papers with <4 pages as they could not contain sufficient information

**Phase 2**

- Inclusion criteria
- Apps designed for general users, existing in both Apple and Android app stores
- Apps that were free to download and could support the English language
- Apps stating the aim as self–health care management
- Apps rated by >10,000 users and having a score of ≥4 out of 5 to ensure that the selected apps were satisfying
- The last update of the current app had to be from January 01, 2018 to ensure that the app was up to date
- Exclusion criteria
- Apps designed for children or people with special needs
- Apps related to an external device such as a smartwatches or shoes
- Apps that did not clarify the date of the last update

**Phase 3**

- Inclusion criteria
- Apps written using either or both Swift or Kotlin
- Open-source apps that supported the English language
- Apps stating the aim as self–health care management
- The source code of the app had to exceed 1000 lines of code
- Exclusion criteria
- Apps designed for children or people with special needs
- Apps related to external devices such as smartwatches or shoes

#### Overview of the Selected Studies

In phase 1, of 52 studies, 27 (52%) were from IEEE, 18 (34%) from SpringerLink, 3 (6%) from ACM, 2 (4%) from PubMed, 1 (2%) from ScienceDirect, and 1 (2%) from Sage. MEDLINE digital data sources were not included as the downloaded papers focused on analyzing user behavior rather than app development, which was outside the scope of this study. In phase 2, we reviewed 21 apps on each platform, each of which had 2 versions: 1 in the Apple App Store and 1 in Android Google Play. In phase 3, a group of 24 open-source apps was reviewed manually and automatically, including 11 (46%) Android and 13 (54%) iOS apps.

In total, we reviewed 41 iOS and 77 Android apps, including 7 (17%) iOS and 45 (58%) Android apps in phase 1, 21 (51%) iOS and 21 (27%) Android apps in phase 2, and 13 (54%) iOS and 11 (46%) Android apps in phase 3. The apps in phase 2 have almost the same functionalities and structures on both platforms. Thus, we use a letter with a number (eg, *A1*) to represent the app name of the 2 versions, and we specify the differences if they are found on each app.

## Results

### Overview

Here, we summarize the findings obtained by reviewing the selected papers and apps based on our RQs. The general context of apps from the data extracted in phases 1, 2, and 3 are presented in Multimedia Appendix 1 [6-81]. The main characteristics of the 3 phases are presented in Multimedia Appendix 2.

### The Characteristics of Self-management mHealth Apps

#### Crucial Functionalities

**Figure 2:**
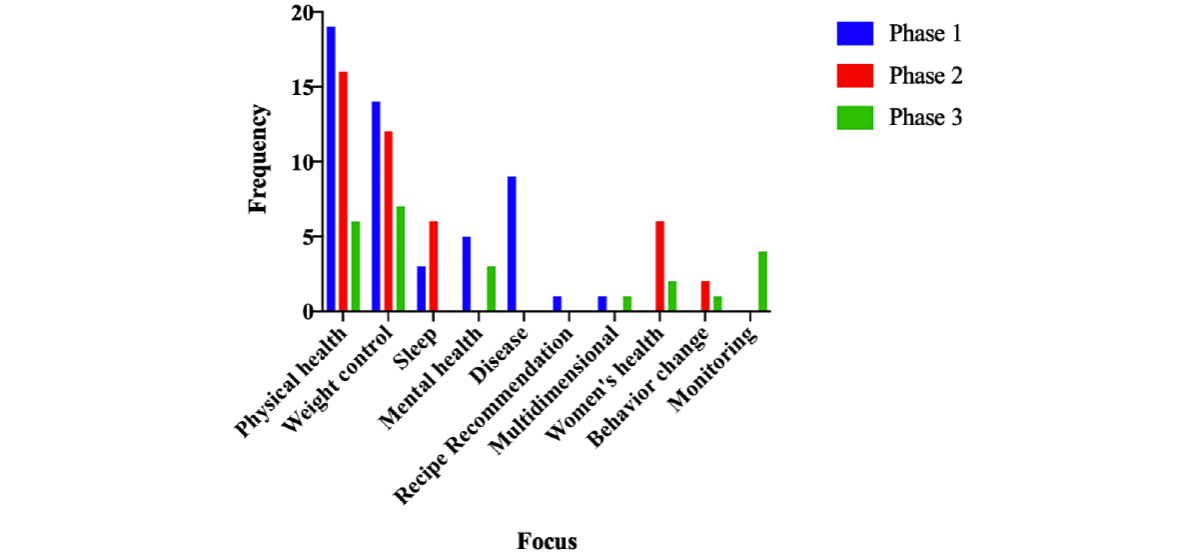
The general focus of reviewed apps.

**Figure 3:**
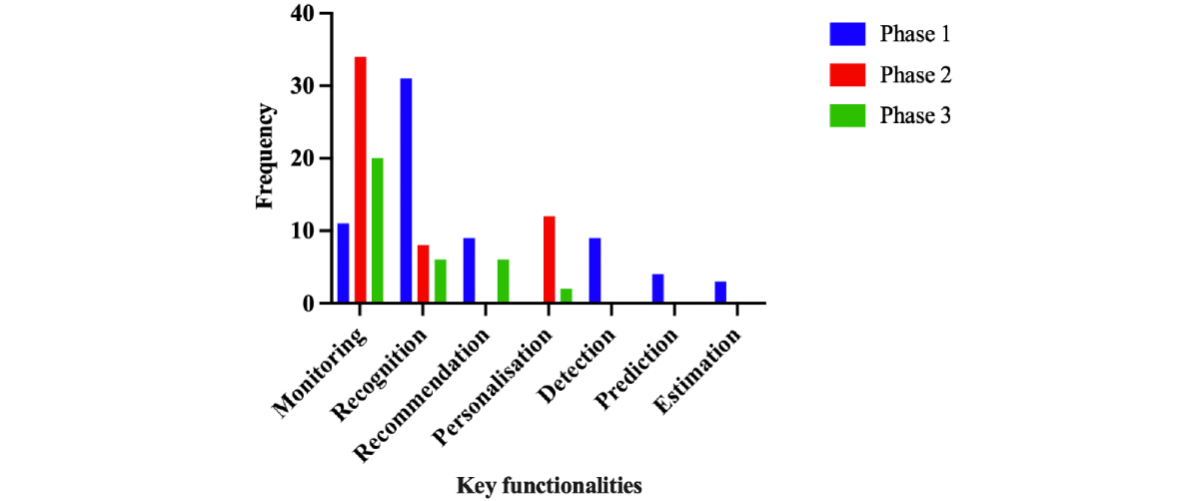
The key functionalities in the 3 phases.

Self-management mHealth apps had several focuses, including physical health, weight control, sleep, mental health, disease, women’s health, and monitoring, as shown in Figure 2, where physical health and weight control were the most frequent focus in the 3 phases. These apps used various terminologies to describe their crucial functionalities, including detection, recognition, prediction, estimation, monitoring, personalization, and recommendations. The word *detection* was used to detect whether there was something abnormal in data, such as a disease, whereas the word *recognition* was used to recognize the type of something, such as the type of specific activity or food. Prediction and estimation can use collected data to predict a situation or estimate a value. Monitoring depends on the calculation methods used to monitor the user’s progress. Regarding personalization and recommendation, the apps provided customized plans, guidelines, and suggestions based on the users’ data and their progress. Figure 3 shows the frequency of studies and apps, including their functionalities in the 3 phases. Specifically, 60% (31/52) of the studies in phase 1 focused on recognition. However, 81% (34/42) of the apps in phase 2 and 83% (20/24) of the apps in phase 3 focused on monitoring. In contrast, a few studies in phase 1 demonstrated the usual app functionalities such as log-in and analysis as they focused on presenting their new contributions in developing ML algorithms. Phases 2 and 3 profusely included usual functionalities, including log-in, payment, synchronization of data from other apps, rating or questionnaire, search, sharing data using email, WhatsApp, and Telegram, and analysis to periodically visualize reports or charts to help users easily read results and achieve their desired goals.

#### UI Components

The most frequent UI components in the 3 phases were labels, images, buttons, input boxes, lists, visualized charts to present user progress, menu or hamburger menu, and radio buttons. Other components such as tables, visual control bars, and virtual pets appeared less frequently. Furthermore, apps in phases 2 and 3 had more frequency of the following UI components than phase 1: switch, scroll bar, picker, calendar, and media.

#### Navigation and Structure

To navigate through an app’s screens, the next and back buttons, tab structure, hamburger menu, and home page could be used. In phase 1, the tab structure appeared in 12% (6/52) of studies. The next and back buttons were used in 10% (5/52) of studies. One of the studies included navigation through both the next and back button and the tab, whereas another study supported navigation with the next and back button, tab pages, and hamburger menu. Approximately 4% (2/52) of studies reported that the user could use the home page to navigate to other pages.

Most apps (40/42, 95%) in phase 2 had, at start-up, multiple simple pages for customization with backward and forward arrows; however, the basic app was presented as a tab structure with multiple tabs. In the case of iOS, of the 42 apps, 18 (43%) were designed with a tab structure, 2 (5%) supported navigation with tabs and a hamburger menu, and the remaining 1 (2%) app had a main page with hamburger menu. For Android, of the 42 apps, 17 (40%) followed the tab structure, 3 (7%) had a page with a hamburger menu, and 1 (2%) had a main page with buttons to navigate to other pages. Consequently, 86% (36/42) of apps had a similar structure on both platforms, where 81% (34/42) of the apps followed the tabbed UI approach, and approximately 5% (2/42) of the apps had a main page with hamburger menu.

In phase 3, 71% (17/24) of apps had a tabbed UI structure, including 33% (8/24) having a tab with a back arrow to navigate to the previous or main page, 29% (7/24) of apps supporting navigation through only tabs, and 8% (2/24) of apps supporting navigation through both tabs and hamburger menu. Approximately 29% (7/24) of apps had a main page structure, including 8% (2/24) supporting a main page with a back arrow, 8% (2/24) having a main page with a back arrow and menu, 8% (2/24) having a main page with navigation buttons to other pages, and 4% (1/24) having a main page with a menu.

#### Services and Technologies

Several local and remote services were used in the reviewed studies and apps. These services included accessing remote and local libraries and technologies; accessing external libraries using an application programming interface (API) such as Clarifai, ZXing (Zebra Crossing), Edamam, Dialogflow, and ToneAnalyzerV3 (IBM Watson); web services; cloud services such as Google’s cloud computing engine (Firebase); linking to other apps such as social media; and Google Maps. In addition, some apps used developer frameworks such as HealthKit, ARKit, SceneKit, and StepCounter.

Many studies and apps in the 3 phases had access to built-in technologies such as cameras, motion sensors (accelerometers and gyroscopes), location (GPS), and microphones. Phases 2 and 3 had access to photographs, vibrations, networks, audio, phones, and storage. These technologies can collect various types of data, including sensor data, images, voice, and text. The collected data can be used to process through ML algorithms to obtain useful information.

Several studies in phase 1 used ML algorithms that differentiated between supervised and unsupervised learning with their branches: classification, regression, clustering, and association. Some studies evaluated multiple algorithms to determine the most accurate algorithm. Most of the investigated studies used supervised learning for classification, including naïve Bayes, support vector machines, logistic regression, K-nearest neighbor, rule-based classifiers, decision trees, ridge, AdaBoost, bagging, Gaussian processes, ensemble of nested dichotomies, rotation forest, Fisher vector representation, linear classifiers, and artificial neural networks (NNs), which included specific types such as deep NNs and deep convolutional NNs. Moreover, supervised learning was used for regression, which comprised linear regression (LR), Bayesian ridge, support vector regression, gradient boosting, and AdaBoost. Some studies used algorithms for both classification and regression, such as random forest. Other studies used unsupervised learning for clustering, such as the density-based spatial clustering of apps with noise and molecular complex detection. Other used algorithms were the kernel null Foley-Sammon transform and t-distributed stochastic neighbor embedding, the threshold method, decision tables, radial basis function kernel, ensemble extreme learning machine, and sequential minimal optimization. In contrast, many studies used measurement methods, including BMI, basal metabolic rate, Gaussian and LR functions, and general calculations.

#### Security Features

Authentication through log-in was the principal security feature in the 3 phases, which was achieved either by creating an app account using email or linking it with other accounts such as Facebook, Apple, or Google. However, only one of the studies (S16) mentioned authentication through log-in in phase 1. This limitation could be because of the focus on building and evaluating a new ML algorithm rather than a complete app. Approximately 95% (40/42) of apps in phase 2 and 42% (10/24) of apps in phase 3 supported log-in. The log-in password was hidden using points or stars.

#### Architectures and Patterns

In phase 1, some studies involved the development of mobile apps to collect real data; however, they analyzed these data on a physical computer, server, or specific tool (eg, Waikato Environment for Knowledge Analysis or MATLAB) to extract features and identify the most accurate ML algorithm. Most of these studies focused on building a suitable model using ML algorithms without providing real information about the mHealth app, which integrates the final model with implementation or architecture. These apps stored the automatically-collected data remotely on a server (2/52, 4%) using a client-server architecture (a web-based mechanism) or locally (7/52, 13%) on a mobile device (an offline mechanism). Approximately 2% (1/52) of studies used pre-existing data sets for processing on a physical computer to build the model. Other studies focused on developing complete self-management mHealth apps, in which 54% (28/52) of studies used a web-based mechanism for developing the apps. They followed a client-server architecture, where a mobile app acts as a client node to receive data and connect to a server (sometimes a cloud server) to process data or perform computations. Other studies (12/52, 23%) used an offline mechanism to process received data locally on mobile devices. One of the studies (S24) in phase 1 followed MobileNetV2 (deep NN architecture). In S13, the authors used cloud-based virtualization (hypervisor architecture), which depends on virtual swaps between mobile sessions to separate the physical resources into virtual resources on a cloud server to use computing power efficiently. Consequently, in phase 1, 58% (30/52) of the studies used a web-based mechanism, and 37% (19/52) used an offline mechanism. In phase 3, 83% (20/24) were web-based apps and 17% (4/24) were offline apps.

Regarding the design pattern, 2 authors (S38 and S40) in phase 1 stated that they used the model-view-controller (MVC) design pattern, whereas, in phase 3, a total of 3 design patterns were used in the explored apps: MVC, model-view-viewmodel, and view-interactor-presenter-entity-router. MVC was used in 75% (18/24) and model-view-viewmodel in 21% (5/24) of the apps. View-interactor-presenter-entity-router was used in 4% (1/24) of apps.

In terms of the architecture for implementing ML mobile apps in phase 1, of the 52 studies, 18 (35%) used web-based inference based on a pretraining model by researchers, 10 (19%) used offline inference on devices based on a pretraining model by researchers, 3 (6%) used both web-based and offline inference based on a pretraining model by researchers, and 2 (4%) used web-based inference based on ready solutions (API), whereas, 29% of ML apps in phase 3 used web-based inference based on ready solutions (API). Web-based and offline inferences were used for the same functionality in S30 and S51, where the web was used to accelerate the process, and offline was used when a connection was lost but with lower performance and high resource consumption. However, S1 used both web-based and offline inferences for different functionalities.

#### Logging Mechanism

Most studies and apps collected data from user information, activities, or behaviors, which could be gathered automatically from sensors, manually by users’ logging, or both. Automatic collection can be achieved either through synchronization and importing of data from other apps or by accessing built-in technologies or developer frameworks such as HealthKit on iOS or Google Fit on Android. Then, the collected data were automatically analyzed using ML algorithms or calculation methods to provide useful feedback and personalized services.

In phase 1, most studies (24/52, 46%) used built-in technologies for automatic data collection. Other studies required manual input of information (8/52, 15%), image capture (14/52, 27%), or voice recording (2/52, 4%). Few studies (4/52, 8%) supported both automatic collection and manual inputs.

In phase 2, manual logging was used in 62% (26/42) of apps and could be performed through barcode scanning, input of personal information, or searching the app’s internal database. Approximately 24% (10/42) of the apps supported automatic logging through built-in technologies or synchronization with other apps. Approximately 14% (6/42) of the apps supported both automatic and manual logging.

Most apps (18/24, 75%) in phase 3 supported manual logging of data such as personal information, food and water consumption, sleep, and emotion. Approximately 17% (4/24) of the apps supported manual logging and synchronization. One of the apps presented real-time information from an external server, and another app supported the automatic collection of data.

#### Development Approach

Prototyping was the most commonly used approach adopted in 15% (8/52) of studies. Other studies used agile (S35), extreme programming (S15), iterative development (S38), or user-centered approaches (S26 and S29). The remaining studies in phase 1 and the other phases did not mention their approach.

#### Operating System and Programming Language

In phase 1, 85% (44/52) of studies targeted Android, forming the majority. Approximately 13% (7/52) supported iOS, and 2% (1/52) targeted both iOS and Android. In terms of operating system (OS) versions, S2 used Android OS version 4.4.2; S6 and S28 used Android OS version 4.1.2; S22 used Android OS version 1.6; S29 used Android OS version 4.2.2 (Jellybean); S41 used Android OS version 2.3; and S39 targeted both platforms, with Android version 4.0 (Ice Cream Sandwich) and iOS version 3.2. The Java programming language was used in S1, S3, S16, S17, S21, S28, S31, S34, S40, and S38. Plain Old Java Objects were used in S42. Each selected app in phase 2 was available on both the Android and iOS OSs. We reviewed and evaluated 24 apps in phase 3, 11 (46%) of which had Android OS and 13 (54%) of which had iOS.

#### Evaluation

Different evaluation techniques were used in phase 1; however, most studies (38/52, 73%) measured the performance of ML algorithms through experiments, pilot studies, or randomized controlled trials and compared the performance with state of the art. Furthermore, some studies used a specific tool to test the accuracy of various classifiers and select the most appropriate one. For example, 12% (6/52) of the studies used the Waikato Environment for Knowledge Analysis, and approximately 6% (3/52) used MATLAB. Furthermore, cross-validation was used in approximately 17% (9/52) of the studies to accurately calculate the performance metrics. Such metrics could include confusion matrix, sensitivity, specificity, and accuracy. Other studies (4/52, 8%) evaluated through a usability study, such as user acceptability and subjective surveys. Approximately 10% (5/52) of the studies combined either or both comparisons with state-of-the-art and usability studies.

In phase 2, we applied a systematic quality evaluation of the apps selected from app stores. The evaluation was conducted by the first author and revised by the second author. Each app was opened on both the Android and iOS platforms for evaluation. We evaluated the apps using MARS, comprising 5 main sections (engagement, functionality, aesthetics, information, and subjective quality), which are presented in Multimedia Appendix 3, where the tables list the scores for each section and the final mean score of every app. The app’s section score was calculated by taking the average score of each item for each app. A2 and A17 received the highest score (4.6) in the engagement section, whereas A10 and A16 received the lowest score (3.2). The mode of the functionality section was 4.25, whereas it was 4.7 in the aesthetics section. The information section had the highest score (4.9) in 2 apps (A3 and A15), whereas the other apps received a score of 4.7.

Table 1 reports the mean scores for an overall score of the quality of each app and the subjective quality. The overall score of the app quality was the average of the section scores (excluding subjective quality, which was calculated separately). The median overall mean score was 4.46/5. A2 and A17 received the highest overall score (4.56/5), whereas A10 had the lowest score (3.83/5). In subjective quality evaluation, A6 and A17 received the highest score (4.25/5), and A13 had the lowest score (2/5).

In phase 3, we evaluated the apps using SonarCloud, as shown in Multimedia Appendix 4. We found that most apps had a relatively small number of lines of code, ranging from 1.1 to 7 K.

**Table 1:** Overall and subjective Mobile App Rating Scale evaluation of self-management mobile health apps.

App ID^a^	Overall score, mean (SD)	Subjective quality, mean (SD)
A1	4.46 (0.27)	3.75 (0.50)
A2	4.56 (0.21)	4 (0.82)
A3	4.06 (0.60)	2.75 (1.26)
A4	4.45 (0.33)	2.75 (1.26)
A5	4.51 (0.22)	4 (0.82)
A6	4.38 (0.44)	4.25 (0.96)
A7	4.14 (0.47)	2.25 (0.96)
A8	4.46 (0.27)	3.25 (1.71)
A9	4.19 (0.51)	2.75 (1.26)
A10	3.83 (0.64)	2.25 (0.96)
A11	4.14 (0.47)	2.25 (0.96)
A12	4.46 (0.27)	3.25 (1.71)
A13	4.45 (0.33)	2 (1.41)
A14	4.14 (0.47)	2.25 (0.96)
A15	4.38 (4.45)	3.25 (1.71)
A16	4.04 (0.63)	3 (1.41)
A17	4.56 (0.21)	4.25 (0.96)
A18	4.32 (0.30)	2.5 (1.0)
A19	4.51 (0.22)	4 (0.82)
A20	4.36 (0.43)	2.75 (1.26)
A21	4.51 (0.22)	2.75 (1.26)

^a^App ID represents the app name of the 2 versions, and we specify the differences if they were found for each app.

### The Challenges and Issues of Self-management mHealth Apps

Some studies in phase 1 mentioned general challenges related to mobile devices and app architecture. The first challenge was the restricted number of resources (10/52, 19%), including computational power, storage capacity, and energy efficiency. Other studies referred to the challenge of dealing with a large variety of mobile devices with different software and hardware, which complicates the development of new algorithms (S28) and causes varying levels of accuracy when data are collected from sensors (S32). Furthermore, S17 mentioned security as a challenge for mobile apps.

In contrast, S23 summarized the drawbacks of cloud-based approaches (a web-based mechanism): latency, privacy, cost, and connectivity. S23 also mentioned the limitations of the offline mechanism that integrates the model in the app, which requires republishing a new version of the app with each update of the model and could be inconvenient for the user and result in a waste of time. S42 mentioned the challenge of designing mHealth apps that succeeded in attracting and sustaining users’ interests.

Other studies encountered difficulties in collecting and dealing with the collected data, such as identifying the position of the device on a user’s body when collecting data in 8% (4/52) of studies; determining the set of collected sensors in S11; extracting efficient features from collected data in 8% (4/52) of studies; accurate recognition of activities in real time in 6% (3/52) of studies; and accurate detection of heart rate in 4% (2/52) of studies, which depends on the lighting conditions and location of the finger on the camera lens.

In terms of image processing, the authors of S45 mentioned the limitations of mobile devices in dealing with complex images for extracting features and classifying algorithms. The authors of S23 and S15 mentioned that the properties of an image might be affected by various factors, such as the angle, brightness, focal distance, and camera resolution. The authors of S15 and S49 stated that food recognition could be difficult because of several factors, such as diversity of food size, form, color, and texture, as well as deformation and segmentation of food elements, which may affect the identification of food type and calculation of quantity and nutritional value.

In phase 2, we found that most apps (26/42, 62%) used manual logging of data, such as manually inputting the type and duration of the exercise, user emotion, and the category and quantity of consumed food, without using ML algorithms such as automatic recognition of activity and food type. In addition, all explored apps targeted general users without experimental or clinical studies to support their safety, reliability, and effectiveness. We also noticed that most apps (26/42, 62%) needed the internet to access some functions, two of which supported offline working through downloading of content. Approximately 33% (14/42) of apps could work without an internet connection; and 5% (2/42) of apps could not work at all without the internet. We also checked whether the installed apps were hybrid or native by activating the developer option on the Android OS from settings and by turning on the layout bounds option. We found that most Android apps (18/21, 86%) were native. However, this method was not applicable to apps developed using react-native or flutter as they convert the language to native app code. Therefore, the number of native apps was not completely accurate. However, it clarifies to some extent that separate implementations need to be written for each platform, which requires additional time, cost, and effort.

Each app in phase 3 was analyzed and evaluated using SonarCloud, as shown in Multimedia Appendix 4. Of the 24 apps, bugs existed in 7 (29%)—1 (4%) in Android and 6 (25%) in iOS. Approximately 17% (4/24) of apps on iOS had vulnerability issues. Of the 24 apps, 23 (96%) on both platforms had code smells. The highest number of code smells was 181, whereas the lowest was 3. Most apps (18/24, 75%) had duplications, ranging from 0.3% to 14%. Furthermore, we tried to run the apps, and of the 24 apps, only 11 (46%) worked, including 7 (29%) Android and 4 (17%) iOS.

## Discussion

### Comparison and Synthesis of Phases

In this section, we compare and synthesize the collected data and findings from the first, second, and third phases to answer our RQs.

By comparing the characteristics and challenges of the 3 phases, we identified that the phases were different in terms of the use of ML, processing techniques, functionalities, inference of ML, logging mechanisms, evaluation techniques, and challenges. However, they were similar in the most frequent focus, UI components, navigation and structure, services and technologies, authentication features, and architecture and patterns. As shown in Tables 2, 3, and 4, we reviewed 52 studies in phase 1, 21 apps in phase 2 (each of which has 2 versions), and 24 apps in phase 3. We found that most studies of phase 1 (43/52, 83%) were intelligent and used ML algorithms, supporting supervised learning (39/52, 75%), unsupervised learning (1/52, 2%), both supervised and unsupervised learning (1/52, 2%), and accessing external ML libraries through API (2/52, 4%). Most supervised learning studies (34/52, 65%) focused on classification. Some studies used ML in phase 2 (14/42, 33%) and phase 3 (7/24, 29%), where phase 3 depended on an external ML library (API).

In terms of processing techniques, most studies from phase 1 used data (25/52, 48%) and image (11/52, 21%) processing, whereas calculation methods were the most used techniques in phase 2 (22/42, 52%) and phase 3 (13/24, 54%). Data processing included sensor data, questionnaire answers, conversation, and specific data such as goals and preferred meals. The calculation depended on specific equations such as BMI. With respect to focus, the 3 phases were similar, with the most frequent focuses being on physical health and weight control. However, they differed for crucial functionalities, where recognition (20/52, 38%) and detection (9/52, 17%) were the most frequent functionalities in phase 1. Monitoring was the most crucial function in phases 2 and 3, representing 52% (22/42) and 54% (13/24), respectively. The 3 phases were almost similar in terms of UI components and navigation. The most commonly used UI components were labels, images, and buttons, and most apps were designed with a tab structure.

Regarding services and technologies, the camera, GPS, motion sensors, and microphones were frequently used in the 3 phases. The motion included access to accelerometers and gyroscopes and had the highest percentage (21/52, 40%) in phase 1, whereas the camera (20/42, 48%) was the most frequent built-in technology in phase 2, and GPS (7/24, 29%) had the highest percentage in phase 3. Apps in all phases used log-in functionality as a security feature for authentication, which could be achieved by creating a new account with the app using a social media account. For architecture and patterns, most studies in phase 1 (30/52, 58%) and apps in phase 3 (20/24, 83%) used client servers. MVC was the only pattern used in phase 1 and the most used pattern in phase 3 (18/24, 75%). However, phase 2 did not contain an architecture section as there was insufficient information about it in the app stores. In terms of inference of ML, many apps (18/52, 35%) in phase 1 used web-based inference using a pretrained model by researchers. Phase 3 concentrated on web-based inference, with 29% (7/24) of apps using ready solutions, such as the IBM Watson API. Furthermore, the prototype was the most commonly used development approach in phase 1. However, phases 2 and 3 did not include the development approach section because of limited information.

Most apps (46%) in phase 1 were distinguished by automating the logging mechanism using built-in technologies and ML algorithms to automatically recognize types, quantities, and calories of food and physical activities such as walking, running, or jumping with burned calories. Apps in phases 2 and 3 (26/42, 62%, and 18/24, 75%, respectively) concentrated on monitoring functionality through manual logging of specific activities such as eating an apple or walking.

We used different techniques for the evaluation. In phase 1, we summarized the evaluation techniques used by the authors of the research papers, where 58% (30/52) evaluated performance. We used MARS evaluation in phase 2 and SonarCloud in phase 3. Thus, the results of the evaluation were different for each phase.

**Table 2:** Characteristics of phase 1 studies (N=52).

Characteristics		Phase 1 studies, n (%)
**Number of surveyed studies or apps**		
	Android	45 (87)
	iOS	6 (12)
	Both	1 (2)
**Roles of ML^a^**		
	Recognition	19 (37)
	Detection	6 (12)
	Prediction	4 (8)
	Recognition and monitoring	4 (8)
	Recognition and recommendation	4 (8)
	Recognition and estimation	2 (4)
	Recommendation and monitoring	1 (2)
	Recommendation	1 (2)
	Estimation	1 (2)
	Recognition, recommendation, and monitoring	1 (2)
**Types of ML**		
	Supervised learning	39 (75)
	Unsupervised learning	1 (2)
	Both	1 (2)
	External ML library	2 (4)
**Processing techniques**		
	Data	25 (48)
	Image	11 (21)
	Image and calculation	4 (8)
	Data and calculation	4 (8)
	Voice	3 (6)
	Calculation	3 (6)
	Image, data, and calculation	2 (4)
**Focus**		
	Physical health	19 (37)
	Weight control	14 (27)
	Disease	9 (17)
	Mental health	5 (10)
	Sleep	3 (6)
	Recipe’s recommendation	1 (2)
	Multidimensional	1 (2)
**Crucial functionalities**		
	Recognition	20 (38)
	Detection	9 (17)
	Prediction	4 (8)
	Recognition and recommendation	4 (8)
	Recognition and monitoring	4 (8)
	Recommendation and monitoring	3 (6)
	Monitoring	3 (6)
	Recognition and estimation	2 (4)
	Estimation	1 (2)
	Recommendation	1 (2)
	Recognition, recommendation, and monitoring	1 (2)
**UI^b^ components**		
	Label	18 (35)
	Image	17 (33)
	Button	15 (29)
	Input box	8 (15)
	List	8 (15)
**Navigation and structure**		
	Tab	6 (12)
	Back and next	5 (10)
	Main page	2 (4)
	Tab and back and next	1 (2)
	Tab, back and next, and hamburger menu	1 (2)
**Services and technologies**		
	Motion sensors	21 (40)
	Camera	18 (35)
	GPS	2 (4)
	Microphone	4 (8)
**Security features**		
	Log-in	1 (2)
**Architectures and patterns**		
	Client-server (web-based)	30 (58)
	On device (offline)	19 (37)
	MVC^c^	2 (4)
**Inference of ML**		
	Web-based inference	18 (35)
	Offline inference	10 (19)
	Both	3 (7)
	Web-ready solutions	2 (4)
**Development approach**		
	Prototype	8 (15)
	User-centered design	2 (4)
	Agile	1 (2)
	Extreme programming	1 (2)
	Iterative	1 (2)
**Logging mechanisms**		
	Automatic	24 (46)
	Manual	24 (46)
	Both	4 (8)
**Evaluation**		
	Algorithm’s performance	30 (58)
	Algorithm’s accuracy	9 (17)
	Algorithm’s performance and cross-validation	8 (15)
	Usability study	4 (8)
	Cross-validation	1 (2)

^a^ML: machine learning.

^b^UI: user interface.

^c^MVC: model-view-controller.

**Table 3:** Characteristics of phase 2 studies (N=42).

Characteristics		Phase 2 studies, n (%)
**Number of surveyed studies or apps**		
	iOS	21 (50)
	Android	21 (50)
**Roles of ML^a^**		
	Recognition, monitoring, and personalization	6 (14)
	Monitoring and personalization	6 (14)
	Recognition	2 (5)
**Processing techniques**		
	Calculation	22 (52)
	Calculation and data	8 (19)
	Calculation, data, and image	6 (14)
	Voice	2 (5)
**Focus**		
	Physical health	16 (38)
	Weight control	12 (29)
	Women’s health	6 (14)
	Sleep	6 (14)
	Behavior change	2 (5)
**Crucial functionalities**		
	Monitoring	22 (52)
	Recognition, monitoring, and personalization	6 (14)
	Monitoring and personalization	6 (14)
	Recognition	2 (5)
**UI^b^ components**		
	Label	42 (100)
	Image	42 (100
	Button	42 (100)
	List	42 (100)
	Scroll bar	42 (100)
	Input box	34 (81)
**Navigation and structure**		
	Tab (iOS)	18 (43)
	Tab (Android)	17 (40)
	Main page and hamburger menu (Android)	3 (7)
	Tab and hamburger menu (iOS)	2 (5)
	Main page and hamburger menu (iOS)	1 (2)
	Main page (Android)	1 (2)
**Services and technologies**		
	Camera	20 (48)
	GPS	26 (62)
	Motion sensors	7 (17)
	Microphone	4 (10)
**Security features**		
	Log-in	40 (95)
**Logging mechanisms**		
	Manual	26 (62)
	Automatic	10 (24)
	Both	6 (14)
**Evaluation**		
	MARS^c^	42 (100)

^a^ML: machine learning.

^b^UI: user interface.

^c^MARS: Mobile App Rating Scale.

**Table 4:** Characteristics of phase 3 studies (N=24).

Characteristics		Phase 3 studies, n (%)
**Number of surveyed studies or apps**		
	iOS	13 (54)
	Android	11 (46)
**Roles of ML^a^**		
	Recommendation and monitoring	2 (8)
	Recognition	2 (8)
	Recognition and recommendation	2 (8)
	Recognition and monitoring	1 (4)
**Types of ML**		
	External ML library	7 (29)
**Processing techniques**		
	Calculation	13 (54)
	Calculation and data	5 (21)
	Data	2 (8)
	Image	1 (4)
	Voice	1 (4)
	Calculation and image	1 (4)
**Focus**		
	Weight control	7 (29)
	Physical health	6 (25)
	Monitoring	4 (17)
	Mental health	3 (13)
	Women’s health	2 (8)
	Behavior change	1 (4)
	Multidimensional	1 (4)
**Crucial functionalities**		
	Monitoring	13 (54)
	Recommendation and monitoring	4 (17)
	Recognition	2 (8)
	Recognition and recommendation	2 (8)
	Recognition and monitoring	1 (4)
	Monitoring and personalization	1 (4)
**UI^b^ components**		
	Label	24 (100)
	Input box	23 (96)
	Image	22 (92)
	Button	22 (92)
	List	16 (67)
**Navigation and structure**		
	Tab (iOS)	11 (46)
	Tab (Android)	4 (17)
	Main page and menu (Android)	3 (13)
	Main page (Android)	3 (13)
	Main page (iOS)	1 (4)
	Tab and hamburger menu (iOS)	1 (4)
	Tab and hamburger menu (Android)	1 (4)
**Services and technologies**		
	GPS	7 (29)
	Camera	5 (21)
	Motion sensors	1 (4)
	Microphone	1 (4)
**Security features**		
	Log-in	10 (42)
**Architectures and patterns**		
	Client-server (web-based)	20 (83)
	On device (offline)	4 (17)
	MVC^c^	18 (75)
	MVVM^d^	5 (21)
	VIPER^e^	1 (4)
**Inference of ML**		
	Web-based inference through ready solutions	7 (29)
**Logging mechanisms**		
	Manual	18 (75)
	Both	4 (17)
	Automatic	2 (8)
**Evaluation**		
	SonarCloud	24 (100)

^a^ML: machine learning.

^b^UI: user interface.

^c^MVC: model-view-controller.

^d^MVVM: model-view-viewmodel.

^e^VIPER: view-interactor-presenter-entity-router.

In terms of challenges and issues, phase 1 mentioned several challenges, such as the restricted number of resources in mobile devices, mobile device fragmentation, security of mobile apps, drawbacks of web-based and offline mechanisms, designing of an attractive and sustainable mobile app, and difficulties in collecting and processing data to design an efficient ML algorithm. Phase 2 reported the importance of network connectivity, which may affect the efficiency of a mobile app in the case of a connection loss. In addition, it highlighted the issues of writing separate implementations for each platform and the absence of care provider involvement in the development and evaluation phases. Of the analyzed apps in phase 3, we found that iOS apps had more bugs than Android apps, which may, however, be because of the developers and not the platform. Most apps on both platforms had code smells, duplications, and performance issues. Only iOS apps had vulnerability issues. Furthermore, the logging mechanisms of the second and third phases were primitive and needed improvement to remain up to date with those described in research papers.

As a result, we found that commercial apps in phase 2 and open-source apps in phase 3 had more common aspects than the apps of research papers in phase 1. They were similar in that most used calculation methods as processing techniques and monitoring as a crucial functionality. In addition, they were simple and complete apps that were partially supported by ML and automatic logging. In contrast, apps in phase 1 were complex and intelligent, although some of them were incomplete, presenting a gap between real and research paper apps.

### Principal Findings

This research involved various studies and apps designed for the general population with the aim of self–health care management. Most of these apps were developed with a specific focus, requiring users to download several apps to cover different aspects. Therefore, multidimensional well-being apps that combine multiple focuses need more research and development as it is better to download a single app with a set of features than to download several apps.

Furthermore, we found that the development of self-management mHealth apps required significant efforts from researchers to build and evaluate new algorithms and from developers to deal with different techniques and frequent updates of apps to stay up to date with the latest technologies. A parent example is developing apps with ML algorithms, which comprise several steps implemented manually, including collecting data, extracting features, and applying several ML algorithms to determine the most accurate algorithm. For example, the authors of S7 compared 7 classifiers: support vector machines, naïve Bayes, K-nearest neighbor, decision trees, LR, NNs, and rule-based classifiers. Furthermore, many other studies manually compared multiple algorithms to find the best algorithm, requiring a long time and great effort from researchers. Another example is the development of the same app in different languages and techniques to be compatible with multiple platforms.

In contrast, mobile devices are handheld gadgets with limited resources (eg, storage, computational power, and battery energy), which significantly hinders the improvement of service qualities such as ML algorithms that require dealing with intensive data and heavy computations. Connection with remote services such as the cloud can address these limitations. Therefore, many apps that used ML algorithms followed a web-based inference to achieve optimal performance within a reasonable time. However, this approach is generally insufficient when the connection is lost and may pose security issues. Therefore, some apps integrated a pretrained model with the mobile app (offline inference), which may cause some difficulty when updating the models. Only 4% (2/52) of apps supported both web-based and offline inferences for the same functionality but with lower performance and high energy consumption. Consequently, many challenges still exist related to finding an adequate algorithm that fulfills the specific requirements of intelligent self-management mHealth apps, as well as an efficient architecture that supports web-based and offline inferences with the ability to reduce resource consumption and execution time and increase performance, specifically when using large ML algorithms.

### Threats to Validity

#### Threats to Internal Validity

##### Instrumental Bias

To ensure the consistency of our evaluation results, all evaluation processes in the second and third phases were performed in the same manner by the first author. The evaluation in phase 2 was applied to the same app on both Android and iOS platforms. Furthermore, the evaluation process of phase 3 was repeated to double check the results.

##### Selection Bias

To ensure that we adopted unbiased and consistent procedures in the selection, we used the quasi–gold standard approach [4], which includes manual and automated search strategies, as well as snowballing. We selected the highest-quality peer-reviewed papers published in 7 web-based digital libraries. We further complemented our research with snowballing to capture as many studies as possible and minimize the potential for missing any relevant studies.

#### Threats to External Validity: Generalization to Different Samples

We reviewed studies that involved the implementation of a research study from 2008 to 2020. However, the generalizability of our findings could be affected by the exclusion of studies that presented theoretical research without implementation, as well as studies and apps linked with external devices.

#### Threats to Construct Validity

The RQs of this review could not entirely cover all of the reviewed research papers and self-management mHealth apps. Some research papers and apps had fewer or more details than the information identified in our RQs.

#### Threats to Content Validity

##### Relevance

To comprehensively identify the characteristics and issues of the selected studies, we divided the review into 3 main phases using different data sources, including research papers, commercial apps from digital Apple and Android app stores, and open-source apps from GitHub.

##### Representativeness

In phases 1 and 3, we selected mobile apps developed with either Kotlin or Swift on the Android and iOS platforms, respectively. These apps had a wide range of functionalities and purposes related to self–health care management.

#### Threats to Conclusion Validity

We extracted data from the assigned studies and self-management mHealth apps from the app stores and GitHub. To ensure the validity and consistency of the extracted data, the protocol for the data extraction strategy and format was developed by the first author and reviewed by the second author. In addition, we created a Microsoft Excel file to record and arrange the extracted data and check their relevance to our RQs.

### Limitations and Future Work

This study had some limitations. The review was prepared and reviewed by 2 authors; however, it would have been better if it had more reviewers. In the second phase, apps were limited to self–health care management and included only those available in both Android and iOS digital app stores. The review would be more comprehensive if phase 2 included other categories of self-management mHealth apps, and phases 1 and 3 included other programming languages, such as Java and Objective-C. Furthermore, the review might have been more generalizable if it included an app designed for children and people with special needs.

Developing mobile apps requires significant effort because of the complexity of self-management mHealth apps. Therefore, we have started the development of a framework that accelerates and facilitates the development of mHealth apps [82,83]. The framework semiautomatically generates Android and iOS mobile apps and will be enhanced with frequent characteristics that resulted from this review, such as tab structure, predefined components of ML algorithms, and local and external services. Moreover, the framework supports both web-based and offline inference, which appears to be a limitation of current self-management mHealth apps that usually support one of them, as the web-based mechanism could lead to unusable apps if the connection is lost, whereas the offline mechanism requires updating the entire app with each algorithm enhancement and library update.

### Comparison With Prior Work

Several SLRs have been conducted on mHealth apps. For example, in the study by Mosa et al [84], the authors classified the functionalities of mHealth apps. They found that smartphones were useful tools for self–health care and clinical communication. Furthermore, smartphones can be used for the remote monitoring of patients, disease self-management, and patient education. In the study by Dounavi and Tsoumani [85], the authors described the effectiveness of mHealth apps in facilitating weight management behaviors by following healthy food consumption and physical activity. They found that mHealth apps are considered easy to use and useful in achieving weight loss because they involve users in the treatment plan, thereby increasing their commitment. These studies focused on mobile apps from a health care perspective. In contrast, our focus was on the software engineering perspective to identify the characteristics and challenges of mHealth apps, helping developers and researchers understand the infrastructure and functional and technical aspects of mHealth apps. Another SLR [86] focused on examining and identifying the empirical usability evaluation processes of mHealth apps. They stated that these processes could be improved by adopting automated mechanisms and combining >1 evaluation method. Furthermore, they demonstrated the importance of adapting mHealth apps according to user requirements.

This study aimed to conduct a comprehensive review and evaluation of self-management mHealth apps. This study gathered empirical evidence from the literature to identify the characteristics and challenges of existing self-management mHealth apps focused on self–health care management. The main contribution of this research is its detailed analysis and synthesis of relevant literature by software engineering researchers to deeply understand state of the art and provide guidance for the development of complex self-management mHealth apps.

### Conclusions

In this research, we presented the details of an SLR on self–health care management mobile apps that consisted of three main phases. The results of this research can serve as a basis for researchers and developers to understand the characteristics of self-management mHealth apps and know the existing challenges that require further research. In phase 1, we reviewed 44 studies published between 2008 and 2020. In phase 2, 42 apps were reviewed and evaluated using the MARS. In phase 3, we reviewed and evaluated, using SonarCloud, 24 open-source apps from GitHub, including both iOS and Android platforms.

The research papers in phase 1 presented many interesting ideas, used different ML algorithms, and supported automatic logging mechanisms. These algorithms were used to process data to automatically recognize physical activities; diagnose diseases; recognize the types, quantities, and calories of food; and predict the user’s emotion. However, the results of phase 1 show the need for optimization of the architecture and algorithm of intelligent self-management mHealth apps to efficiently include web-based and offline inferences, reduce resource consumption, and increase performance.

In phases 2 and 3, we found that most apps in app stores and GitHub focused on monitoring and analysis functionalities that use calculation methods to create progress reports and charts. However, the quantity of food consumed, exercise, and emotions should be entered manually. They lack automatic recognition of the type and quantity of food or activities. As a result, some advanced features exist in research papers but not in app stores and open-source apps, which may indicate that these features are still under research and development. Subsequently, the apps of phases 2 and 3 might need some improvement to keep pace with the advancement of research.

## Appendix

Multimedia Appendix 1 Context of mobile health apps.

Multimedia Appendix 2 Characteristics of mobile health apps.

Multimedia Appendix 3 Mobile App Rating Scale evaluation of phase 2.

Multimedia Appendix 4 SonarCloud evaluation of phase 3.

## Abbreviations

API: application programming interface
LR: logistic regression
MARS: Mobile App Rating Scale
mHealth: mobile health
ML: machine learning
MVC: model-view-controller
NN: neural network
OS: operating system
RQ: research question
SLR: systematic literature review
UI: user interface

## References

[1] Kitchenham B, Charters S (2007). Guidelines for performing systematic literature reviews in software engineering. Keele University and Durham University Joint Report.

[2] Stoyanov SR, Hides L, Kavanagh DJ, Zelenko O, Tjondronegoro D, Mani M (2015). Mobile app rating scale: a new tool for assessing the quality of health mobile apps. JMIR Mhealth Uhealth.

[3] Sonarcloud.

[4] Zhang H, Babar MA, Tell P (2011). Identifying relevant studies in software engineering. Inf Softw Technol.

[5] (2008). iPhone app store downloads top 10 million in first weekend. Apple Newsroom.

[6] Li H, Zhang Q, Lu K (2015). Integrating mobile sensing and social network for personalized health-care application. Proceedings of the 30th Annual ACM Symposium on Applied Computing.

[7] Jia B, Li J (2014). Recognizing human activities in real-time using mobile phone sensors. Proceedings of the 8th China Conference on Advances in Wireless Sensor Networks.

[8] Guo J, Zhou X, Sun Y, Ping G, Zhao G, Li Z (2016). Smartphone-based patients' activity recognition by using a self-learning scheme for medical monitoring. J Med Syst.

[9] Wang C, Xu Y, Zhang J, Yu W (2016). COPO: a novel position-adaptive method for smartphone-based human activity recognition. Proceedings of the 10th Asia-Pacific Services Computing Conference on Advances in Services Computing.

[10] Prudêncio J, Aguiar A, Lucani D (2013). Physical activity recognition from smartphone Embedded Sensors. Proceedings of the 6th Iberian Conference on Pattern Recognition and Image Analysis.

[11] Lu Y, Wei Y, Liu L, Zhong J, Sun L, Liu Y (2016). Towards unsupervised physical activity recognition using smartphone accelerometers. Multimed Tools Appl.

[12] Shoaib M, Scholten H, Havinga PJ (2013). Towards physical activity recognition using smartphone sensors. Proceedings of the IEEE 10th International Conference on Ubiquitous Intelligence and Computing and IEEE 10th International Conference on Autonomic and Trusted Computing.

[13] Cao L, Wang Y, Jin Q, Ma J (2017). ActiRecognizer: design and implementation of a real-time human activity recognition system. Proceedings of the 2017 International Conference on Cyber-Enabled Distributed Computing and Knowledge Discovery.

[14] Chen Z, Jiang C, Xie L (2019). A novel ensemble ELM for human activity recognition using smartphone sensors. IEEE Trans Ind Inf.

[15] Beily MD, Badjowawo MD, Bekak DO, Dana S (2016). A sensor based on recognition activities using smartphone. Proceedings of the 2016 International Seminar on Intelligent Technology and Its Applications.

[16] Saha J, Chowdhury C, Biswas S (2017). Device independent activity monitoring using smart handhelds. Proceedings of the 7th International Conference on Cloud Computing, Data Science & Engineering.

[17] Ravì D, Lo B, Yang GZ (2015). Real-time food intake classification and energy expenditure estimation on a mobile device. Proceedings of the IEEE 12th International Conference on Wearable and Implantable Body Sensor Networks.

[18] Peddi SV, Yassine A, Shirmohammadi S (2015). Cloud based virtualization for a calorie measurement e-health mobile application. Proceedings of the 2015 IEEE International Conference on Multimedia & Expo Workshops.

[19] Villalobos G, Almaghrabi R, Pouladzadeh P, Shirmohammadi S (2012). An image processing approach for calorie intake measurement. Proceedings of the 2012 IEEE International Symposium on Medical Measurements and Applications.

[20] Ocay AB, Fernandez JM, Palaoag TD (2017). NutriTrack: Android-based food recognition app for nutrition awareness. Proceedings of the 3rd IEEE International Conference on Computer and Communications.

[21] Tangsripairoj S, Wongkham N, Leelalerkiat B, Chuenpukdi S (2019). WhatTheHealth: an Android application for consumers of healthy food. Proceedings of the 16th International Joint Conference on Computer Science and Software Engineering.

[22] Kumbhar D, Patil S (2017). Mobile cloud based system recognizing nutrition and freshness of food image. Proceedings on the 2017 International Conference on Energy, Communication, Data Analytics and Soft Computing.

[23] Montanini L, Sabino N, Spinsante S, Gambi E (2018). Smartphone as unobtrusive sensor for real-time sleep recognition. Proceedings of the 2018 IEEE International Conference on Consumer Electronics.

[24] Yamashita Y, Onodera M, Shimoda K, Tobe Y (2019). Emotion-Polarity Visualizer on Smartphone. Proceedings of the IEEE 27th International Requirements Engineering Conference Workshops.

[25] Shrestha A, Won M (2018). DeepWalking: enabling smartphone-based walking speed estimation using deep learning. Proceedings of the 2018 IEEE Global Communications Conference.

[26] Matsumura K, Yamakoshi T (2013). iPhysioMeter: a new approach for measuring heart rate and normalized pulse volume using only a smartphone. Behav Res Methods.

[27] Pelegris P, Banitsas K, Orbach T, Marias K (2010). A novel method to detect heart beat rate using a mobile phone. Annu Int Conf IEEE Eng Med Biol Soc.

[28] Dai X, Spasić I, Meyer B, Chapman S, Andres F (2019). Machine learning on mobile: an on-device inference app for skin cancer detection. Proceedings of the 4th International Conference on Fog and Mobile Edge Computing.

[29] Ech-Cherif A, Misbhauddin M, Ech-Cherif M (2019). Deep neural network based mobile dermoscopy application for triaging skin cancer detection. Proceedings of the 2nd International Conference on Computer Applications & Information Security.

[30] Wadhawan T, Situ N, Lancaster K, Yuan X, Zouridakis G (2011). SkinScan©: a portable library for melanoma detection on handheld devices. Proc IEEE Int Symp Biomed Imaging.

[31] Ghandeharioun A, McDuff D, Czerwinski M, Rowan K (2019). EMMA: an emotion-aware wellbeing chatbot. Proceedings of the 8th International Conference on Affective Computing and Intelligent Interaction.

[32] Jiménez-Serrano S, Tortajada S, García-Gómez JM (2015). A mobile health application to predict postpartum depression based on machine learning. Telemed J E Health.

[33] Figueiredo IN, Leal C, Pinto L, Bolito J, Lemos A (2016). Exploring smartphone sensors for fall detection. mUX J Mob User Exp.

[34] Bhatia A, Kumar S, Mago V (2015). Gradient: a user-centric lightweight smartphone based standalone fall detection system. Proceedings of the 17th Portuguese Conference on Artificial Intelligence.

[35] Sanabria P, Benedetto JI, Neyem A, Navon J, Poellabauer C (2018). Code offloading solutions for audio processing in mobile healthcare applications: a case study. Proceedings of the IEEE/ACM 5th International Conference on Mobile Software Engineering and Systems.

[36] Khan NS, Muaz MH, Kabir A, Islam MN (2017). Diabetes predicting mHealth application using machine learning. Proceedings of the 2017 IEEE International WIE Conference on Electrical and Computer Engineering.

[37] Kelly D, Curran K, Caulfield B (2017). Automatic prediction of health status using smartphone-derived behavior profiles. IEEE J Biomed Health Inform.

[38] Fahim M, Vui LB, Fatima I, Lee S, Yoon Y (2013). A sleep monitoring application for u-lifecare using accelerometer sensor of smartphone. Proceedings of the 7th International Conference on Ubiquitous Computing and Ambient Intelligence: Context-Awareness and Context-Driven Interaction.

[39] Sandulescu V, Andrews S, Ellis D, Dobrescu R, Martinez-Mozos O (2015). Mobile app for stress monitoring using voice features. Proceedings of the 2015 E-Health and Bioengineering Conference.

[40] Kalaiselvan V, Azman F, Cheng LK, Rahim FA (2019). nocturnOWL: sleep-monitoring virtual pet mobile application. Proceedings of the 2019 IEEE Conference on Open Systems.

[41] Lomaliza JP, Park H (2017). A highly efficient and reliable heart rate monitoring system using smartphone cameras. Multimed Tools Appl.

[42] Yang PC, Su SC, Wu IL, Chiang JH (2015). Life Record: a smartphone-based daily activity monitoring system. Proceedings of the 6th International Conference on Advances in Swarm and Computational Intelligence.

[43] Villarreal V, Otero M (2016). Development a mobile system based on the harris-benedict equation to indicate the caloric intake. Proceedings of the 10th International Conference on Ubiquitous Computing and Ambient Intelligence.

[44] de la Torre Díez I, Garcia-Zapirain B, López-Coronado M, Rodrigues JJ, Del Pozo Vegas C (2017). A new mHealth app for monitoring and awareness of healthy eating: development and user evaluation by Spanish users. J Med Syst.

[45] Sánchez CH, Ramírez MR, Palencia JS, Lobato BY, del Carmen Osuna Millán N (2017). My health info: an informative mHealth app for healthcare and weight control. Proceedings of the 5th KES International Conference on Innovation in Medicine and Healthcare.

[46] Harries T, Eslambolchilar P, Stride C, Rettie R, Walton S (2013). Walking in the wild - using an always-on smartphone application to increase physical activity. Proceedings of the 14th IFIP TC 13 International Conference on Human-Computer Interaction.

[47] Rahman F, Henninger P, Kegley D, Sullivan K, Yoo J (2018). Healthy hankerings: motivating adolescents to combat obesity with a mobile application. Proceedings of the 20th International Conference on Human-Computer Interaction. Interaction in Context.

[48] Zhao Z, Sun Z, Huang L, Guo H, Wang J, Xu H (2016). iRun: a smartphone-based system to alert runners to warm up before running. Proceedings of the 11th International Conference on Wireless Algorithms, Systems, and Applications.

[49] Fahim M, Baker T, Khattak AM, Alfandi O (2017). Alert me: enhancing active lifestyle via observing sedentary behavior using mobile sensing systems. Proceedings of the IEEE 19th International Conference on e-Health Networking, Applications and Services.

[50] Kong F, He H, Raynor HA, Tan J (2015). DietCam: multi-view regular shape food recognition with a camera phone. Pervasive Mob Comput.

[51] Yang S, Zhou P, Duan K, Hossain MS, Alhamid MF (2018). emHealth: towards emotion health through depression prediction and intelligent health recommender system. Mobile Netw Appl.

[52] Zhang W, Yu Q, Siddiquie B, Divakaran A, Sawhney H (2015). "Snap-n-Eat": food recognition and nutrition estimation on a smartphone. J Diabetes Sci Technol.

[53] Maruyama T, Kawano Y, Yanai K (2012). Real-time mobile recipe recommendation system using food ingredient recognition. Proceedings of the 2nd ACM International Workshop on Interactive Multimedia on Mobile and Portable Devices.

[54] Duan P, Wang W, Zhang W, Gong F, Zhang P, Rao Y (2013). Food image recognition using pervasive cloud computing. Proceedings of the 2013 IEEE International Conference on Green Computing and Communications and IEEE Internet of Things and IEEE Cyber, Physical and Social Computing.

[55] Tran DN, Phan DD (2016). Human activities recognition in Android smartphone using support vector machine. Proceedings of the 7th International Conference on Intelligent Systems, Modelling and Simulation.

[56] Zhu F, Bosch M, Woo I, Kim S, Boushey CJ, Ebert DS, Delp EJ (2010). The use of mobile devices in aiding dietary assessment and evaluation. IEEE J Sel Top Signal Process.

[57] Lane ND, Lin M, Mohammod M, Yang X, Lu H, Cardone G, Ali S, Doryab A, Berke E, Campbell AT, Choudhury T (2014). BeWell: sensing sleep, physical activities and social interactions to promote wellbeing. Mobile Netw Appl.

[58] (2020). Calcheck Health App. GitHub.

[59] (2020). Corona Tracker Awareness App. GitHub.

[60] (2020). Easy Healthy Diet. GitHub.

[61] (2019). Eat Healthy App. GitHub.

[62] (2019). Health Care. GitHub.

[63] (2020). Health Diary. GitHub.

[64] (2020). jHealth. GitHub.

[65] (2020). LindaJamii. GitHub.

[66] (2020). Period. GitHub.

[67] (2020). QuitSmoking. GitHub.

[68] (2021). SCD-Food-List-Android. GitHub.

[69] (2021). ActiveBoomers. GitHub.

[70] (2019). BodyMindfulness. GitHub.

[71] (2020). CalorieCounter. GitHub.

[72] (2020). Fitness. GitHub.

[73] (2019). Health4Food. GitHub.

[74] (2018). HealthAppYouFit-Ios. GitHub.

[75] (2020). HealthMate. GitHub.

[76] (2020). IOS-HealthTracker. GitHub.

[77] (2019). Healthy-Diet. GitHub.

[78] (2019). iHealth. GitHub.

[79] (2018). mHealthApp. GitHub.

[80] (2019). Moodify. GitHub.

[81] (2019). smARt_pet. GitHub.

[82] Alwakeel L, Lano K (2020). Model driven development of mobile application. King's College London.

[83] Lano K, Alwakeel L, Rahimi SK, Haughton H (2021). Synthesis of mobile applications using AgileUML. Proceedings of the 14th Innovations in Software Engineering Conference (formerly known as India Software Engineering Conference).

[84] Mosa AS, Yoo I, Sheets L (2012). A systematic review of healthcare applications for smartphones. BMC Med Inform Decis Mak.

[85] Dounavi K, Tsoumani O (2019). Mobile health applications in weight management: a systematic literature review. Am J Prev Med.

[86] Zapata BC, Fernández-Alemán JL, Idri A, Toval A (2015). Empirical studies on usability of mHealth apps: a systematic literature review. J Med Syst.

