# Supplementary material for: Functional and Technical Aspects of Self-management mHealth Apps: Systematic App Search and Literature Review
Source: JMIR Hum Factors. 2022 May 25;9(2):e29767. doi: 10.2196/29767 (PMC9178446; doi:10.2196/29767)
Supplement: Multimedia Appendix 1 [file humanfactors_v9i2e29767_app1.docx]

**Multimedia Appendix 1. (Context of mHealth Apps)**

Table 1. The general context of mHealth apps (Phase1).

| **Study ID** | **Ref.** | **Processing Techniques** | **General Focus** | **Operating System** | **Architecture of Machine Learning Inference** |
| --- | --- | --- | --- | --- | --- |
| S1 | [6] | Data processing for recognition and recommendation | Physical health | Android | Online and offline inference based on pretrained model by researcher |
| S2 | [7] | Data processing for recognition | Physical health | Android | Offline inference based on pretrained model by researcher |
| S3 | [8] | Data processing for recognition | Physical health | Android | Online inference based on pretrained model by researcher |
| S4 | [9] | Data processing for recognition | Physical health | Android | Developing ML algorithm without mobile app |
| S5 | [10] | Data processing for recognition | Physical health | Android | Developing ML algorithm without mobile app |
| S6 | [11] | Data processing for recognition | Physical health | Android | Developing ML algorithm without mobile app |
| S7 | [12] | Data processing for recognition | Physical health | Android | Developing ML algorithm without mobile app |
| S8 | [13] | Data processing for recognition | Physical health | Android | Online inference based on pretrained model by researcher |
| S9 | [14] | Data processing for recognition | Physical health | Android | Developing ML algorithm without mobile app |
| S10 | [15] | Data processing for recognition | Physical health | Android | Online inference based on pretrained model by researcher |
| S11 | [16] | Data processing for recognition | Physical health | Android | Online inference based on pretrained model by researcher |
| S12 | [17] | Image processing for recognition, data processing for recognition, and calculation methods for estimation | Weight control | Android | Offline inference based on pretrained model by researcher |
| S13 | [18] | Image processing for recognition and calculation methods for monitoring | Weight control | Android | Online inference based on pretrained model by researcher |
| S14 | [19] | Image processing for recognition and calculation methods for monitoring | Weight control | iOS | Online inference based on pretrained model by researcher |
| S15 | [20] | Image processing for recognition and calculation methods for monitoring | Weight control | Android | Online inference based on Clarifai API |
| S16 | [21] | Data processing for recommendation, calculation methods for monitoring and barcode recognition | Weight control | Android | Online inference based on ZXing API |
| S17 | [22] | Image processing for recognition | Weight control | Android | Online inference based on pretrained model by researcher |
| S18 | [23] | Data processing for recognition | Sleep | Android | Developing ML algorithm without mobile app |
| S19 | [24] | Voice processing for recognition | Mental health | Android | Online inference on server using lexical and phonetic analysis to calculate scores  (Measurements without ML) |
| S20 | [25] | Data processing for estimation | Physical health | Android | Developing ML algorithm without mobile app |
| S21 | [26] | Image processing for detection | Disease (measuring heartbeat rate and normalized pulse volume) | iOS | Offline inference using measurement of flash LED and camera (measurements without ML) |
| S22 | [27] | Image processing for detection | Disease (measuring heartbeat rate) | Symbian, Android, and Windows | Offline inference using measurement of flash and camera (Measurements without ML) |
| S23 | [28] | Image processing for detection | Disease | iOS | Offline inference based on pretrained model by researcher |
| S24 | [29] | Image processing for detection | Disease | iOS | Offline inference based on pretrained model by researcher |
| S25 | [30] | Image processing for detection | Disease | iOS | Offline inference based on pretrained model by researcher |
| S26 | [31] | Data processing for recognition | Mental health | Android | Online inference based on pretrained model by researcher |
| S27 | [32] | Data processing for recognition | Mental health | Android | Offline inference based on pretrained model by researcher |
| S28 | [33] | Data processing for detection | Physical health | Android | Developing ML algorithm without mobile app |
| S29 | [34] | Data processing for detection | Physical health | Android | Developing ML algorithm without mobile app |
| S30 | [35] | Voice processing for prediction | Disease | Android | Online and offline inference based on pretrained model by researcher |
| S31 | [36] | Data processing for prediction | Disease | Android | Offline inference based on pretrained model by researcher |
| S32 | [37] | Data processing for prediction | Disease | Android | Online inference based on pretrained model by researcher |
| S33 | [38] | Data processing for recognition and calculation methods for monitoring | Sleep | Android | Online inference based on pretrained model by researcher |
| S34 | [39] | Voice processing for detection | Mental health | Android | Offline inference based on pretrained model by researcher |
| S35 | [40] | Data processing for recommendation and calculation methods for monitoring | Sleep | Android | Recommendation based on measurement (without ML) |
| S36 | [41] | Image processing for detection | Disease (measuring heartbeat rate) | Android | Offline inference using measurement of flash LED and camera (without ML) |
| S37 | [42] | Data processing for recognition | Physical health | Android | Online inference based on pretrained model by researcher |
| S38 | [43] | Calculation methods for monitoring | Weight control | Android | - |
| S39 | [44] | Data processing for recommendation and calculation methods for monitoring | Weight control | Android & iOS | Recommendation based on measurement (without ML) |
| S40 | [45] | Calculation methods for monitoring | Weight control | Android | - |
| S41 | [46] | Calculation methods for monitoring | Physical health | Android | - |
| S42 | [47] | Data processing for recommendation | Weight control | Android | Online inference based on pretrained model by researcher |
| S43 | [48] | Data processing for recognition and recommendation | Physical health | Android | Developing ML algorithm without mobile app |
| S44 | [49] | Data processing for recognition and recommendation | Physical health | Android | Offline inference based on pretrained model by researcher |
| S45 | [50] | Image processing for recognition | Weight control | iOS | Online inference based on pretrained model by researcher |
| S46 | [51] | Data processing for prediction | Mental health | Android | Online inference based on pre-training data by researcher |
| S47 | [52] | Image processing for recognition and estimation | Weight control | Android | Online inference based on pretrained model by researcher |
| S48 | [53] | Image processing for recognition and recommendation | Recipe Recommendation | Android | Online inference based on pretrained model by researcher |
| S49 | [54] | Image processing for recognition | Weight control | Android | Online inference based on pretrained model by researcher |
| S50 | [55] | Data processing for recognition | Physical health | Android | Online inference based on pretrained model by researcher |
| S51 | [56] | Image processing for recognition | Weight control | Android | Online and offline inference based on pretrained model by researcher |
| S52 | [57] | Data processing for recommendation and calculation methods for monitoring | Multi-dimensional wellbeing (Physical health, Sleep, and Mental health) | Android | Offline inference based on pretrained model by researcher |

Table 2 (Part 1). The general context of mHealth apps (Phase 2).

| **App ID ^a^** | **App name** | **Average Rating ^b^** | **Upgrade price** | **Developer** | **Affiliations** | **App Development** |
| --- | --- | --- | --- | --- | --- | --- |
| A1 | Calorie Counter – MyFitnessPal | 4.55 | 39.99£/Year; 7.99/Month | Under Armour, Inc. | Commercial | Native |
| A2 | Flo My Health & Period Tracker | 4.8 | 44.99£/Year | FLO Health, Inc. | Commercial | Native |
| A3 | One You Couch to 5K | 4.75 | - | Public Health England | Government | Native |
| A4 | Fastic Fasting App & Intermittent Fasting Tracker | 4.75 | 2.52£/Week (Annually); 1.95£/Week (Quarterly); 2.52£/Week (Month) | HealthVida GmbH & Co. KG. | Commercial | Hybrid |
| A5 | Calorie Counter by Lose It! For Diet & Weight Loss | 4.65 | 29.99£/Year | FitNow | Commercial | Native |
| A6 | Clue Period Tracker, Ovulation & Cycle Calendar | 4.75 | 3.99£/Month; 23.99£/Year; 19.99£/6Month | BioWink GmbH | Commercial | Native |
| A7 | Period Tracker; Period Calendar Ovulation Tracker | 4.9 | 39.99£/Year | ABISHKKING Limited | Commercial | Native |
| A8 | Zero - Simple Fasting Tracker | 4.7 | 9.99£/Month; 67.99£/Year | Big Sky Health | Commercial | Native |
| A9 | Pacer Pedometer: Walking, Running, Step Challenges | 4.7 | 29.49£/Year; 4.99£/Month; 99.99£/LifeTime | Pacer Health, Inc. | Commercial | Native |
| A10 | Carb Manager: Keto Diet Tracker & Macros Counter | 4.75 | 8.49£/Month; 15.49£/3Months; 38.99£/Year | WombatApps LLC | Commercial | Hybrid |
| A11 | 5K Runner: 0 to 5K in 8 Weeks. Couch potato to 5K | 4.75 | 9.99£/Lifetime | FITNESS22 LTD | Commercial | Native |
| A12 | Relax Melodies: Sleep Sounds | 4.65 | 59.99£/Year | Ipnos Software | Commercial | Native |
| A13 | Smoke Free: Quit Smoking Now and Stop for good | 4.7 | 6.99£/Month; 29.99£/Year | David Crane | Commercial | Native |
| A14 | Relive: Run, Ride, Hike & more | 4.7 | 6.99£/Month; 38.99£/Year | Relive B.V. | Commercial | Native |
| A15 | Insight Timer - Meditation, Sleep, Music | 4.8 | 55.99£/Year | Insight Network Inc. | Commercial | Hybrid |
| A16 | StepsApp Pedometer | 4.65 | 2.99£/Lifetime | StepsApp GmbH | Commercial | Native |
| A17 | Seven - 7 Minute Workout | 4.55 | 9.99£/Month; 59.99£/Year | Perigee | Commercial | Native |
| A18 | Sleep Cycle: Sleep analysis & Smart alarm clock | 4.5 | 24.99£/Year | Sleep Cycle AB | Commercial | Native |
| A19 | Lifesum - Diet & Food Diary | 4.5 | 5.49£/Month; 14.49£/3Month; 24.99£/Year | Lifesum AB | Commercial | Native |
| A20 | Nike Training Club - Home workout & fitness plans | 4.45 | - | Nike, Inc | Commercial | Native |
| A21 | Runkeeper - GPS Track Run Walk | 4.6 | 7.99£/Month; 29.99£/Year | FitnessKeeper, Inc. | Commercial | Native |

^a^ App ID represents the app name of the two versions and we specify the differences if they found on each app.

^b^ Average rating of Android & iOS

Table 2 (Part 2). The general context of mHealth apps (Phase 2).

| **App ID ^a^** | **Processing Techniques** | **General Focus** | **Content** | **Theoretical Background/strategies** |
| --- | --- | --- | --- | --- |
| A1 | Data processing for personalisation, calculation methods for monitoring, and barcode recognition | Weight control | Pictures, videos, and articles | Monitoring/Tracking; Advice /Tips/ Strategies /Skills training |
| A2 | Calculation methods for monitoring | Women health | Pictures, videos, audio, and articles | Monitoring/Tracking |
| A3 | Calculation methods for monitoring | Physical health | Pictures, videos, audio, and articles | Goal setting; Advice /Tips /Strategies /Skills training |
| A4 | Data processing for personalisation and calculation methods for monitoring | Weight control | Pictures | Monitoring/Tracking; Advice /Tips /Strategies /Skills training |
| A5 | Data processing for personalisation, calculation methods for monitoring, and bar recognition | Weight control | Pictures and articles | Monitoring/Tracking; Advice /Tips /Strategies /Skills training |
| A6 | Calculation methods for monitoring | Women health | Pictures and articles | Monitoring/Tracking |
| A7 | Calculation methods for monitoring | Women health | Pictures, videos, audio, and articles | Monitoring/Tracking |
| A8 | Data processing for personalisation and calculation methods for monitoring | Weight control | Pictures, videos, audio, and articles | Monitoring/Tracking; Advice /Tips /Strategies /Skills training |
| A9 | Calculation methods for monitoring | Physical health | Pictures, videos, and audio | Monitoring/Tracking; Advice /Tips /Strategies /Skills training |
| A10 | Data processing for personalisation and calculation methods for monitoring | Weight control | Pictures and articles | Monitoring/Tracking; Advice /Tips /Strategies /Skills training |
| A11 | Calculation methods for monitoring | Physical health | Pictures and audio | Goal setting; Advice /Tips /Strategies /Skills training |
| A12 | - | Sleep | Pictures and audio | Mindfulness/Meditation |
| A13 | Calculation methods for monitoring | Behaviour change | Pictures | Monitoring/Tracking; Behavioural (positive events) |
| A14 | Calculation methods for monitoring | Physical health | Pictures | Monitoring/Tracking; Advice /Tips /Strategies /Skills training |
| A15 | - | Sleep | Pictures and audio | Mindfulness/Meditation |
| A16 | Calculation methods for monitoring | Physical health | Pictures | Monitoring/Tracking |
| A17 | Data processing for personalisation and calculation methods for monitoring | Physical health | Pictures and videos | Monitoring/Tracking; Advice /Tips /Strategies /Skills training |
| A18 | Voice processing for recognition | Sleep | Pictures | Monitoring/Tracking |
| A19 | Data processing for personalisation, calculation methods for monitoring, and barcode recognition | Weight control | Pictures and articles | Monitoring/Tracking; Advice /Tips /Strategies /Skills training |
| A20 | Calculation methods for monitoring | Physical health | Pictures and videos | Monitoring/Tracking; Advice /Tips /Strategies /Skills training |
| A21 | Calculation methods for monitoring | Physical health | Pictures and audio | Monitoring/Tracking; Advice /Tips /Strategies /Skills training |

^a^ App ID represents the app name of the two versions and we specify the differences if they found on each app.

Table 3. The general context of mHealth apps (Phase 3).

| **App ID** | **Ref.** | **App Name** | **Processing Techniques** | **General Focus** | **Operating System** | **Architecture of Machine Learning Inference** | **App Size (LOC)** |
| --- | --- | --- | --- | --- | --- | --- | --- |
| G1 | [58] | CalCheckHealth | Calculation methods for monitoring | Weight control | Android | - | K2.1 |
| G2 | [59] | CoronaTracker-AwarenessApp | Calculation methods for monitoring | Real-time monitoring (COVID-19) | Android | - | K4.6 |
| G3 | [60] | EasyHealthyDiet | Data processing for recommendation and calculation methods for monitoring | Weight control | Android | Online inference based on Edamam REST API | K4.3 |
| G4 | [61] | EatHealthyApp | Calculation methods for monitoring and image processing for barcode recognition | Weight control | Android | Online inference based on Vision package API from google play service | K2.6 |
| G5 | [62] | HealthCareApp | Data processing for recognition (DialogFlow) | Mental health | Android | Online inference based on Dialogflow API from Google | 3.5K |
| G6 | [63] | HealthDiary | Calculation methods for monitoring | Health Monitoring | Android | - | 1.7K |
| G7 | [64] | jHealth | Data processing for recommendation and calculation methods for monitoring | Multi-dimensional wellbeing (Physical health, Sleep, and Weight control) | Android | Recommendation based on measurement (without ML) | 4.8K |
| G8 | [65] | LindaJamii | Calculation methods for monitoring | Women health | Android | - | 6.6K |
| G9 | [66] | Period | Calculation methods for monitoring | Women health | Android | - | 1.5K |
| G10 | [67] | QuitSmoking | Calculation methods for monitoring | Behaviour change | Android | - | 7K |
| G11 | [68] | SCDFoodList | - | Weight control | Android | - | 1.1K |
| G12 | [69] | ActiveBoomers | Data processing for recommendation and calculation methods for monitoring | Physical health | iOS | Online inference based on Amazon web service (AWS) | 2.7K |
| G13 | [70] | BodyMindfulness | Calculation methods for monitoring | Physical health | iOS | - | 1.1K |
| G14 | [71] | CalorieCounter | Calculation methods for monitoring | Physical health | iOS | - | 1.3K |
| G15 | [72] | FitnessApp | Data processing for personalisation and calculation methods for monitoring | Weight control | iOS | Personalisation based on quiz (without ML) | 2.7K |
| G16 | [73] | Health4Food | Barcode recognition | Weight control | iOS | Online inference based on Nutritionix API | 1.8K |
| G17 | [74] | HealthAppYouFit | Data processing for recommendation and calculation methods for monitoring | Physical health | iOS | Recommendation based on measurement (without ML) | 1.5K |
| G18 | [75] | HealthMate | Calculation methods for monitoring | Health Monitoring | iOS | - | 2.4K |
| G19 | [76] | HealthTracker | Calculation methods for monitoring | Health Monitoring | iOS | - | 1.2K |
| G20 | [77] | HealthyDiet | Calculation methods for monitoring | Weight control | iOS | - | 1.4K |
| G21 | [78] | iHealth | Calculation methods for monitoring | Physical health | iOS | - | 1.9K |
| G22 | [79] | mHealthApp | Calculation methods for monitoring | Physical health | iOS | - | 3.5K |
| G23 | [80] | Moodify | Data processing for recognition and recommendation | Mental health | iOS | Online inference based on IBM Watson API | 1.6K |
| G24 | [81] | SmartPet | Voice processing for recognition and recommendation | Mental health | iOS | Online inference based on IBM Watson API | 2.1K |
