# Supplementary material for: Functional and Technical Aspects of Self-management mHealth Apps: Systematic App Search and Literature Review
Source: JMIR Hum Factors. 2022 May 25;9(2):e29767. doi: 10.2196/29767 (PMC9178446; doi:10.2196/29767)
Supplement: Multimedia Appendix 2 [file humanfactors_v9i2e29767_app2.docx]

**Multimedia Appendix 2. (Characteristics of mHealth Apps)**

Table 1. Crucial functionalities and studies that used the functionalities on three phases

| **Crucial functionality** | **Frequency** | **Phase 1** | **Phase 2** | **Phase 3** |
| --- | --- | --- | --- | --- |
| Recognition of physical activities, emotion, food, freshness of food, barcode, conversation and/or sleep | 31  8  6 | S1, S2, S3, S4, S5, S6, S7, S8, S9, S10, S11, S12, S13, S14, S15, S16, S17, S18, S19, S26, S27, S33,  S37, S43, S44, S45, S47, S48, S49, S50, and S51 | (iOS and Android)  A1, A5, A18, and A19 | G4, G5, G15, G16, G23, and G24 |
| Detection of skin disease, heart disease, or fall | 9  0  0 | S21, S22, S23, S24, S25, S28, S29, S34, and S36 | - | - |
| Prediction of health status or emotion | 4  0  0 | S30, S31, S32, and S46 | - | - |
| Recommendation of physical activities, emotion, food, recipes, and/or sleep | 9  0  6 | S1, S16, S35, S39, S42, S43, S44, S48, and S52 | - | G3, G7, G12, G17, G23, and G24 |
| Monitoring of steps, calories, BMI, health, women health, physical activities, emotion, food, sleep, behaviour and/or number of COVID-19 cases | 11  34  20 | S13, S14, S15, S16,  S33, S35, S38, S39, S40, S41, and S52 | (iOS and Android)  A1, A2, A3, A4, A5, A6, A7, A8, A9, A10, A11, A13, A14, A16, A19, A20, and A21 | G1, G2, G3, G4, G6, G7, G8, G9, G10, G11, G12, G13, G14, G15, G17, G18, G19, G20, G21, and G22 |
| Estimation of energy expenditure, walking speed, or nutrition | 3  0  0 | S12, S20, and S47 | - | - |
| Personalisation | 0  12  2 | - | (iOS and Android)  A1, A4, A5, A8, A10, and A19 | G11 and G15 |
| Login | 2  20  10 | S16, and S44 | (iOS and Android)  A1, A2, A3, A4, A5, A6, A7, A8, A9, A10, A12, A13, A14, A15, A16, A17, A18, A19, A20, and A21 | G1, G3, G5, G8, G9, G13, G15, G17, G20, and G22 |
| Payment | 0  38  2 | - | (iOS and Android)  A1, A2, A4, A5, A6, A7, A8, A9, A10, A11, A12, A13, A14, A15, A16, A17, A18, A19, and A21 | G4 and G5 |
| Analysis by creating progress report, chart, and/or statistics | 7  34  12 | S16, S19, S21, S32, S37, S41, and S44 | (iOS and Android)  A1, A2, A4, A5, A6, A7, A8, A9, A10, A13, A14, A16, A17, A18, A19, A20, and A21 | G1, G2, G4, G6, G7, G10, G12, G14, G15, G17, G18, G19, G20, G21, G23, and G24 |
| Synchronising and importing data through connecting to other apps such as Apple health, and Fitbit | 0  34  3 | - | (iOS and Android)  A1, A4, A5, A6, A7, A8, A9, A10, A11, A14, A15, A16, A17, A18, A19, A20, and A21 | G1, G21, and G22 |
| Reminder | 0  34  0 | - | (iOS and Android)  A1, A2, A3, A4, A5, A6, A7, A8, A9, A10, A11, A12, A15, A18, A19, A20, and A21 | - |
| App community including follow friends, comment, like, and join forums | 0  30  0 | - | (iOS and Android)  A1, A2, A4, A5, A7, A8, A9, A10, A11, A12, A13, A14, A15, A17, and A21 | - |
| Achievement, challenge, and mission | 0  22  0 | - | (iOS and Android)  A1, A3, A5, A8, A9, A11, A13, A14, A17, A20, and A21 | - |
| Countdown timer | 0  20  2 | - | (iOS and Android)  A2, A3, A4, A6, A7, A8, A11, A12, A13, and A15 | G10 and G17 |
| Search | 0  20  8 | - | (iOS and Android)  A1, A2, A5, A8, A9, A10, A14, A15, A17, and A19 | G1, G8, G11, G12, G13, G16, G17, and G20 |
| Sharing and exporting through email or social media (Facebook, Instagram, WhatsApp, Messenger, Message, etc.) | 0  20  4 | - | (iOS and Android)  A1, A8, A10, A11, A12, A13, A15, A16, A20, and A21 | G2, G18, G21, and G22 |
| Rating/Questionnaire | 0  16  1 | - | (iOS and Android)  A3, A7, A8, A11, A15, A16, A19, and A21 | G12 |
| Media player | 0  14  0 | - | (iOS and Android)  A3, A11, A12, A15, A18, A20, A21 | - |

Table 2. The UI components of mHealth apps and studies that used the components on three phases.

| **UI component** | **Frequency** | **Phase 1** | **Phase 2** | **Phase 3** |
| --- | --- | --- | --- | --- |
| Label | 18  All  All | S15, S16, S19, S23, S24, S25, S27, S31, S32, S38, S39, S40, S42, S44, S46, S47, S48, and S52 | (iOS and Android)  All | All |
| Image | 17  All  22 | S12, S16, S21, S23, S24, S25, S27, S30, S32, S38, S39, S40, S42, S44, S46, S48, and S52 | (iOS and Android)  All | G1, G2, G3, G4, G5, G7, G8, G9, G10, G12, G13, G14, G15, G16, G17, G18, G19, G20, G21, G22, G23, and G24 |
| Button | 15  All  22 | S2, S3, S13, S15, S16, S21, S26, S27, S31, S35, S38, S39, S40, S42, and S44 | (iOS and Android)  All | G1, G2, G3, G4, G5, G6, G7, G8, G9, G10, G12, G13, G14, G15, G16, G17, G18, G19, G20, G22, G23, and G24 |
| Input box | 8  34  23 | S2, S3, S15, S26, S27, S38, S40, and S42 | (iOS and Android)  A1, A2, A4, A5, A7, A8, A9, A10, A12, A13, A14, A15, A17, A18, A19, A20, and A21 | G1, G2, G3, G4, G5, G6, G7, G8, G9, G10, G11, G12, G13, G14, G15, G16, G17, G18, G19, G20, G22, G23, and G24 |
| List | 8  All  16 | S12, S15, S30, S31, S38, S42, S46, and S48 | (iOS and Android)  All | G1, G2, G3, G5, G6, G9, G10, G11, G12, G13, G15, G16, G17, G19, G21, and G22 |
| Visual charts and/or counter | 7  40  16 | S16, S19, S21, S32, S37, S41, and S44 | (iOS and Android)  A1, A2, A3, A4, A5, A6, A7, A8, A9, A10, A11, A13, A14, A15, A16, A17, A18, A19, A20, and A21 | G1, G2, G4, G6, G7, G10, G12, G14, G15, G17, G18, G19, G20, G21, G23, and G24 |
| Menu / Hamburger menu | 6  8  5 | S3, S7, S16, S28, S31, and S38 | (iOS)  A2, A11, and A19  (Android)  A2, A11, A19, A20, and A21 | G1, G2, G3, G8, and G24 |
| Radio button | 5  18  2 | S19, S25, S38, S40, and S44 | (iOS and Android)  A2, A4, A11, A14, A15, A17, A18, A19, and A20 | G2 and G7 |
| Table | 2  0  1 | S39 and S40 | - | G13 |
| Visual control bar | 1  16  0 | S30 | (iOS and Android)  A5, A7, A8, A10, A12, A13, A15, and A19 | - |
| Switch (toggle button) | 1  36  6 | S36 | (iOS and Android)  A1, A2, A4, A5, A6, A7, A8, A9, A11, A12, A13, A14, A15, A16, A17, A19, A20, and A21 | G6, G18, G19, G20, G21, and G22 |
| Check box | 1  22  2 | S38 | (iOS and Android)  A2, A3, A6, A7, A9, A10, A12, A15, A19, A20, and A21 | G10 and G11 |
| Scroll bar | 1  All  11 | S48 | (iOS and Android)  All | G2, G3, G4, G7, G9, G10, G11, G12, G17, G18, and G21 |
| Virtual pet | 1  0  1 | S35 | - | G24 |
| Tool bar | 0  36  0 | - | (iOS and Android)  A1, A2, A4, A5, A6, A8, A9, A10, A11, A13, A14, A15, A16, A17, A18, A19, A20, and A21 | - |
| Picker/Spinner | 0  34  11 | - | (iOS and Android)  A1, A2, A4, A5, A7, A8, A9, A10, A11, A13, A15, A16, A17, A18, A19, A20, and A21 | G1, G3, G8, G9, G13, G15, G16, G18, G19, G20, and G22 |
| Calendar | 0  14  3 | - | (iOS and Android)  A2, A5, A6, A7, A10, A16 and A17 | G9, G12, and G23 |
| Media | 0  8  3 | - | (iOS and Android)  A11, A18, A20, and  A21 | G2, G12, and G13 |
| Dialog box | 0  0  3 | - | - | G5, G7 and G11 |

Table 3. The built-in technologies of mHealth apps and studies that used the components on three phases.

| **Built-in technology** | **Frequency** | **Phase 1** | **Phase 2** | **Phase 3** |
| --- | --- | --- | --- | --- |
| Camera | 18  20  5 | S12, S13, S14, S15, S16, S17, S21, S22, S23, S24, S25, S36, S42, S45, S47, S48, S49, and S51 | (iOS and Android)  A1, A5, A8, A9, A10, A11, A14, A15, A20, and A21 | G2, G5, G8, G9, and G22 |
| Accelerometer and Gyroscope  (Motion Sensors) | 21  7  1 | S1, S2, S3, S4, S5, S6, S7, S8, S9, S10, S11, S18, S20, S28, S29, S32, S37, S41, S43, S50, and S52 | (iOS)  A1, A2, A6, A9, A16, A18, and A21 | G22 |
| Location (GPS) | 2  26  7 | S26 and S52 | (iOS and Android)  A1, A2, A3, A8, A9, A11, A12, A13, A14, A16, A18, A20, and A21 | G2, G4, G5, G12, G17, G22, and G24 |
| Microphone | 4  4  1 | S18, S19, S30, and S52 | (iOS and Android)  A15, and A18 | G24 |
| Flash LED (light) | 4  4  0 | S18, S21, S22 and S36 | (iOS and Android)  A1 and A9 | - |
| Photos | 0  2  3 | - | (iOS)  A1 and A14 | G10, G14, and G24 |
| HealthKit, CareKitUI ARKit, SceneKit, and/or Step counter and step detector | 0  4  6 | - | (iOS)  A1, A4, A9, and A16 | G1, G14, G17, G21, G22, and G24 |
| Vibration | 0  24  2 | - | (iOS and Android)  A1, A4, A5, A6, A7, A8, A12, A14, A15, A17, A18, and A20 | G4 and G10 |
| Network | 0  21  20 | - | (Android)  All | G1, G2, G3, G4, G5, G8, G9, G10, G12, G13, G14, G15, G16, G17, G18, G20, G21, G22, G23, and G24 |
| Audio or record audio | 0  2  2 | - | (Android)  A15 and A18 | G22 and G5 |
| Phone | 0  5  2 | - | (Android)  A1, A9, A10, A20, and A21 | G2 and G8 |
| Storage | 0  17  3 | - | (Android)  A1, A3, A4, A5, A6, A7, A8, A9, A10, A11, A12, A14, A15, A16, A19, A20, and A21 | G1, G2, and G10 |
| Contacts | 0  8  0 | - | (Android)  A1, A5, A7, A9, A17, A19, A20, and A21 | - |
| Bluetooth | 0  7  0 | - | (Android)  A1, A3, A5, A15, A19, A20, and A21 | - |
| Gravity | 3  0  0 | S2, S3, and S29 | - | - |

Table 4. The ML algorithm of mHealth apps (Phase1).

| **Machine Learning Algorithm** | **Learning Algorithm** | **Frequency** | **Studies that used the Algorithm** |
| --- | --- | --- | --- |
| Support vector machines (SVM) | Supervised Learning for Classification | 15 | S5, S10, S11, S14, S25, S27, S28, S32, S33, S34, S46, S47, S48, S50, and S51 |
| Naive bayes |  | 9 | S7, S8, S10, S11, S17, S26, S27, S31, and S43 |
| K-nearest neighbour (KNN) |  | 8 | S1, S7, S8, S11, S18, S43, S44 and S49 |
| Decision trees |  | 6 | S1, S7, S10, S11, S18, and S42 |
| Logistic regression |  | 3 | S11, S26, S27, and S52 |
| Artificial neural networks |  | 3 | S5, S7, and S27 |
| Deep neural networks |  | 2 | S13 and S30 |
| Deep convolutional neural network |  | 2 | S20 and S24 |
| Bagging |  | 2 | S1 and S26 |
| Gaussian processes |  | 2 | S9 and S26 |
| Convolutional neural network |  | 1 | S23 |
| Rule-based classifiers |  | 1 | S7 |
| Ridge |  | 1 | S26 |
| AdaBoost |  | 1 | S43 |
| Ensemble of nested dichotomies |  | 1 | S29 |
| Rotation forest |  | 1 | S1 |
| Fischer vector representation |  | 1 | S12 |
| Linear regression (classifiers) |  | 1 | S12 and S52 |
| Bayesian |  | 1 | S45 |
| Two-layered classification |  | 1 | S37 |
| Linear regression, Bayesian Ridge, Support vector regression, Gradient boosting, and AdaBoost | Supervised Learning for Regression | 1 | S26 |
| Random forest | Supervised Learning for Classification and Regression | 5 | S2, S3, S4, S18, and S26 |
| Density-based spatial clustering of applications with noise (DBSCAN) | Unsupervised Learning for Clustering | 1 | S3 |
| Molecular complex detection (MCODE) | Unsupervised Learning for Clustering | 1 | S6 |
