# Supplementary material for: Functional and Technical Aspects of Self-management mHealth Apps: Systematic App Search and Literature Review
Source: JMIR Hum Factors. 2022 May 25;9(2):e29767. doi: 10.2196/29767 (PMC9178446; doi:10.2196/29767)
Supplement: Multimedia Appendix 3 [file humanfactors_v9i2e29767_app3.docx]

**Multimedia Appendix 3. (MARS Evaluation of Phase 2)**

Table 1. Engagement of mHealth apps based on MARS.

| **App ID** | **Entertainment** | **Interest** | **Customisation** | **Interactivity** | **Target group** | **Average score** |
| --- | --- | --- | --- | --- | --- | --- |
| A1 | 4 | 4 | 4 | 4 | 5 | 4.2 |
| A2 | 4 | 5 | 5 | 4 | 5 | 4.6 |
| A3 | 3 | 3 | 3 | 3 | 5 | 3.4 |
| A4 | 5 | 5 | 3 | 4 | 5 | 4.4 |
| A5 | 4 | 5 | 4 | 4 | 5 | 4.4 |
| A6 | 4 | 5 | 4 | 4 | 5 | 4.4 |
| A7 | 3 | 3 | 3 | 4 | 5 | 3.6 |
| A8 | 4 | 4 | 4 | 4 | 5 | 4.2 |
| A9 | 5 | 4 | 3 | 4 | 5 | 4.2 |
| A10 | 2 | 2 | 3 | 4 | 5 | 3.2 |
| A11 | 3 | 3 | 4 | 3 | 5 | 3.6 |
| A12 | 4 | 5 | 4 | 3 | 5 | 4.2 |
| A13 | 5 | 5 | 3 | 4 | 5 | 4.4 |
| A14 | 4 | 4 | 2 | 3 | 5 | 3.6 |
| A15 | 4 | 4 | 4 | 3 | 5 | 4 |
| A16 | 3 | 3 | 2 | 3 | 5 | 3.2 |
| A17 | 5 | 5 | 4 | 4 | 5 | 4.6 |
| A18 | 3 | 4 | 4 | 4 | 5 | 4 |
| A19 | 4 | 5 | 4 | 4 | 5 | 4.4 |
| A20 | 5 | 3 | 3 | 3 | 5 | 3.8 |
| A21 | 5 | 4 | 4 | 4 | 5 | 4.4 |

Table 2. Functionality of mHealth apps based on MARS.

| **App ID** | **Performance** | **Ease of use** | **Navigation** | **Gestural design** | **Average score** |
| --- | --- | --- | --- | --- | --- |
| A1 | 5 | 4 | 5 | 3 | 4.25 |
| A2 | 5 | 4 | 5 | 3 | 4.25 |
| A3 | 5 | 4 | 4 | 3 | 4 |
| A4 | 5 | 4 | 4 | 3 | 4 |
| A5 | 5 | 4 | 5 | 3 | 4.25 |
| A6 | 5 | 4 | 3 | 3 | 3.75 |
| A7 | 5 | 4 | 5 | 3 | 4.25 |
| A8 | 5 | 4 | 5 | 3 | 4.25 |
| A9 | 4 | 4 | 3 | 3 | 3.5 |
| A10 | 5 | 4 | 3 | 3 | 3.75 |
| A11 | 5 | 4 | 5 | 3 | 3.25 |
| A12 | 5 | 4 | 5 | 3 | 4.25 |
| A13 | 4 | 4 | 5 | 3 | 4 |
| A14 | 5 | 4 | 5 | 3 | 4.25 |
| A15 | 5 | 4 | 4 | 3 | 4 |
| A16 | 5 | 4 | 5 | 3 | 4.25 |
| A17 | 5 | 4 | 5 | 3 | 4.25 |
| A18 | 5 | 4 | 5 | 3 | 4.25 |
| A19 | 5 | 4 | 5 | 3 | 4.25 |
| A20 | 5 | 4 | 5 | 3 | 4.25 |
| A21 | 5 | 4 | 5 | 3 | 4.25 |

Table 3. Aesthetics of mHealth apps based on MARS.

| **App ID** | **Layout** | **Graphics** | **Visual appeal** | **Average score** |
| --- | --- | --- | --- | --- |
| A1 | 5 | 5 | 4 | 4.67 |
| A2 | 5 | 5 | 4 | 4.67 |
| A3 | 4 | 5 | 3 | 4 |
| A4 | 5 | 5 | 4 | 4.67 |
| A5 | 5 | 5 | 4 | 4.67 |
| A6 | 5 | 5 | 4 | 4.67 |
| A7 | 4 | 5 | 3 | 4 |
| A8 | 5 | 5 | 4 | 4.67 |
| A9 | 4 | 5 | 4 | 4.33 |
| A10 | 3 | 5 | 3 | 3.67 |
| A11 | 4 | 5 | 3 | 4 |
| A12 | 5 | 5 | 4 | 4.67 |
| A13 | 5 | 5 | 4 | 4.67 |
| A14 | 4 | 5 | 3 | 4 |
| A15 | 5 | 5 | 4 | 4.67 |
| A16 | 4 | 5 | 3 | 4 |
| A17 | 5 | 5 | 4 | 4.67 |
| A18 | 5 | 5 | 3 | 4.33 |
| A19 | 5 | 5 | 4 | 4.67 |
| A20 | 5 | 5 | 4 | 4.67 |
| A21 | 5 | 5 | 4 | 4.67 |

Table 4. Information of mHealth apps based on MARS.

| **App ID** | **Accuracy** **^a^** | **Goals** | **Quality of information** | **Quantity of information** | **Visual information** | **Credibility** | **Evidence base** | **Average score** |
| --- | --- | --- | --- | --- | --- | --- | --- | --- |
| A1 | 5 | 6 | 6 | 6 | 6 | 3 | 1 | 4.71 |
| A2 | 5 | 6 | 6 | 5 | 6 | 4 | 1 | 4.71 |
| A3 | 5 | 6 | 6 | 6 | 6 | 4 | 1 | 4.86 |
| A4 | 5 | 6 | 6 | 6 | 6 | 3 | 1 | 4.71 |
| A5 | 5 | 6 | 6 | 6 | 6 | 3 | 1 | 4.71 |
| A6 | 5 | 6 | 6 | 6 | 6 | 3 | 1 | 3.71 |
| A7 | 5 | 6 | 6 | 6 | 6 | 3 | 1 | 4.71 |
| A8 | 5 | 6 | 6 | 6 | 6 | 3 | 1 | 4.71 |
| A9 | 5 | 6 | 6 | 6 | 6 | 3 | 1 | 4.71 |
| A10 | 5 | 6 | 6 | 6 | 6 | 3 | 1 | 4.71 |
| A11 | 5 | 6 | 6 | 6 | 6 | 3 | 1 | 4.71 |
| A12 | 5 | 6 | 6 | 6 | 6 | 3 | 1 | 4.71 |
| A13 | 5 | 6 | 6 | 6 | 6 | 3 | 1 | 4.71 |
| A14 | 5 | 6 | 6 | 6 | 6 | 3 | 1 | 4.71 |
| A15 | 5 | 6 | 6 | 6 | 6 | 4 | 1 | 4.86 |
| A16 | 5 | 6 | 6 | 6 | 6 | 3 | 1 | 4.71 |
| A17 | 5 | 6 | 6 | 6 | 6 | 3 | 1 | 4.71 |
| A18 | 5 | 6 | 6 | 6 | 6 | 3 | 1 | 4.71 |
| A19 | 5 | 6 | 6 | 6 | 6 | 3 | 1 | 4.71 |
| A20 | 5 | 6 | 6 | 6 | 6 | 3 | 1 | 4.71 |
| A21 | 5 | 6 | 6 | 6 | 6 | 3 | 1 | 4.71 |

^a^ Accuracy of the app in description of app store

Table 5. Subjective quality of mHealth apps based on MARS.

| **App ID** | **Recommendation ^a^** | **Usage ^b^** | **Payment ^c^** | **Overall rating ^d^** | **Average score** |
| --- | --- | --- | --- | --- | --- |
| A1 | 4 | 4 | 3 | 4 | 3.75 |
| A2 | 5 | 4 | 3 | 4 | 4 |
| A3 | 4 | 3 | 1 | 3 | 2.75 |
| A4 | 4 | 3 | 1 | 3 | 2.75 |
| A5 | 5 | 4 | 3 | 4 | 4 |
| A6 | 5 | 4 | 3 | 5 | 4.25 |
| A7 | 3 | 2 | 1 | 3 | 2.25 |
| A8 | 5 | 3 | 1 | 4 | 3.25 |
| A9 | 3 | 3 | 1 | 4 | 2.75 |
| A10 | 3 | 3 | 1 | 2 | 2.25 |
| A11 | 3 | 2 | 1 | 3 | 2.25 |
| A12 | 5 | 3 | 1 | 4 | 3.25 |
| A13 | 4 | 1 | 1 | 2 | 2 |
| A14 | 3 | 2 | 1 | 3 | 2.25 |
| A15 | 5 | 3 | 1 | 4 | 3.25 |
| A16 | 4 | 3 | 1 | 4 | 3 |
| A17 | 5 | 4 | 3 | 5 | 4.25 |
| A18 | 3 | 3 | 1 | 3 | 2.5 |
| A19 | 5 | 4 | 3 | 4 | 4 |
| A20 | 3 | 3 | 1 | 4 | 2.75 |
| A21 | 3 | 3 | 1 | 4 | 2,75 |

^a^ Would you recommend this app to people who might benefit from it?

^b^ How many times do you think you would use this app in the next 12 months if it was relevant to you?

^c^ Would you pay for this app?

^d^ What is your overall star rating of the app?
