# Supplementary material for: Functional and Technical Aspects of Self-management mHealth Apps: Systematic App Search and Literature Review
Source: JMIR Hum Factors. 2022 May 25;9(2):e29767. doi: 10.2196/29767 (PMC9178446; doi:10.2196/29767)
Supplement: Multimedia Appendix 4 [file humanfactors_v9i2e29767_app4.docx]

**Multimedia Appendix 4. (SonarCloud Evaluation of Phase 3)**

Table 1. The issues of mHealth apps.

| **App ID** | **Bugs** | **Vulnerability** | **Code smell** | **Duplication** |
| --- | --- | --- | --- | --- |
| G1 | 0 | 0 | 68 | 5.4% |
| G2 | 3 | 0 | 40 | 2.3% |
| G3 | 0 | 0 | 13 | 1.7% |
| G4 | 0 | 0 | 17 | 1.3% |
| G5 | 0 | 0 | 30 | 0.6% |
| G6 | 0 | 0 | 0 | 0% |
| G7 | 0 | 0 | 3 | 0% |
| G8 | 0 | 0 | 7 | 0.3% |
| G9 | 0 | 0 | 18 | 3.6% |
| G10 | 0 | 0 | 45 | 1.9% |
| G11 | 0 | 0 | 11 | 0% |
| G12 | 6 | 1 | 50 | 1.8% |
| G13 | 0 | 0 | 9 | 2.5% |
| G14 | 0 | 0 | 27 | 3.2% |
| G15 | 1 | 0 | 109 | 1.7% |
| G16 | 0 | 0 | 43 | 3.7% |
| G17 | 12 | 1 | 110 | 13.8% |
| G18 | 8 | 0 | 181 | 12.9% |
| G19 | 0 | 0 | 45 | 0% |
| G20 | 0 | 0 | 32 | 1.6% |
| G21 | 0 | 0 | 25 | 0% |
| G22 | 1 | 0 | 75 | 13.6% |
| G23 | 0 | 1 | 66 | 0% |
| G24 | 3 | 2 | 38 | 0.9% |
